# ER Stress Ire1‐Xbp1s Pathway Maintains Youthful Epidermal Basal Layer Through the Regulation of Cell Proliferation

**DOI:** 10.1111/acel.70258

**Published:** 2025-10-12

**Authors:** Daniel Semmy, Kota Abe, Mizuki Honda, Hiroko Omori, Shohei Ogamino, Tobias Clausen Mercurio, Kyosuke Asakawa, Emi K. Nishimura, Shinya Oki, Yasuyuki Ohkawa, Tohru Ishitani

**Affiliations:** ^1^ Department of Homeostatic Regulation, Research Institute of Microbial Diseases Osaka University Osaka Japan; ^2^ Laboratory of Molecular and Cellular Physiology, Graduate School of Integrated Sciences for Life Hiroshima University Hiroshima Japan; ^3^ Central Instrumentation Laboratory, Research Institute of Microbial Diseases Osaka University Osaka Japan; ^4^ Division of Aging and Regeneration, Institute of Medical Science The University of Tokyo Tokyo Japan; ^5^ Division of Functional Genomics, Institute of Resource Development and Analysis Kumamoto University Kumamoto Japan; ^6^ Division of Transcriptomics Medical Institute of Bioregulation Kyushu University Fukuoka Japan; ^7^ Center for Infectious Disease Education and Research (CiDER) Osaka Japan; ^8^ Japan Agency for Medical Research and Development – Core Research for Evolutional Science and Technology (AMED‐CREST) Osaka University Osaka Japan

**Keywords:** cell proliferation, endoplasmic reticulum stress, Ire1 protein, skin aging, unfolded protein response

## Abstract

The endoplasmic reticulum (ER) stress‐response is an adaptive cellular mechanism activated by an accumulation of unfolded proteins within the ER. Although recent evidence shows that the ER stress‐response is activated in aged tissues, and therefore ER stress is considered a candidate driver of aging, the spatiotemporal regulation and roles of the ER stress‐response during aging remain unclear. To address this research gap, we introduced an Ire1‐Xbp1s ER stress‐response pathway‐sensitive reporter into the ultra‐short‐lived vertebrate *Nothobranchius furzeri* that allows for the analysis of its aging processes within a short period of time. Using this reporter in *N. furzeri* , we confirmed the previously reported age‐dependent activation of ER stress in various tissues and identified an unexpected role of the Ire1‐Xbp1s ER stress‐response pathway in regulating epidermal tissue homeostasis and aging. The Ire1‐Xbp1s ER stress‐response pathway is active in the young epidermal basal layer but declines with aging. Photo‐isolation chemistry‐based spatial transcriptomics and functional assays revealed that the Ire1‐Xbp1s pathway maintains young epidermal cell proliferation by activating the cell cycle regulator Vcp, whereas the age‐dependent decline in glucose metabolism reduces Ire1‐Xbp1s activity, consequently downregulating cell proliferation. Collectively, our study elucidates a previously unidentified role of the ER stress‐response in skin aging, which can offer insights into therapeutic targets for promoting healthy skin.

## Graphical abstract

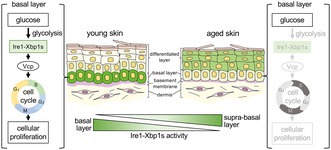

## 1 Introduction

In several multicellular organisms, stem cells maintain tissue integrity through proliferation (Brunet et al. 2023). Specifically, in the skin, a stratified epithelium that protects organisms from insults and infections, stem cells in the basal layer of the epidermis can self‐renew and play essential roles in constant cell turnover and wound repair (Simpson et al. 2011). During aging, epidermal stem cells undergo exhaustion and lose their proliferative capacity, which leads to a decline in their ability to maintain tissue homeostasis and repair damaged tissues (Goodell and Rando 2015; Liu et al. 2019; Inomata et al. 2009). Although the mechanisms controlling epidermal stem cell activity and skin aging have been extensively studied, it is not well understood how intrinsic cellular signaling changes affect cell proliferation during aging.

In addition to stem cell exhaustion, the loss of proteostasis is a hallmark of tissue aging (López‐Otín et al. 2013). Proteostasis refers to the system that maintains protein homeostasis within cells by overseeing protein synthesis, ensuring protein quality control, and employing adaptive mechanisms to mitigate the accumulation of unfolded and misfolded proteins, thereby preventing abnormal protein aggregation and supporting tissue homeostasis (Hipp et al. 2019). The ubiquitin‐proteasome and lysosome‐autophagy systems are major players in proteostasis maintenance, eliminating unfolded proteins from cells through proteolysis, and the activities of both systems decrease with aging (Kaushik and Cuervo 2015). In addition to cytosolic proteostasis, the endoplasmic reticulum (ER) stress‐response system also contributes to proteostasis in organelles. ER plays a central role in the synthesis and correct folding of approximately one‐third of the cellular proteome (Braakman and Bulleid 2011). Under conditions such as hypoxia, nutrient deprivation, increased protein oxidation, or disruptions in secretory pathways, the ER may excessively accumulate misfolded proteins, leading to a condition known as ER stress (Walter and Ron 2011). ER stress activates three canonical pathways to prevent misfolded protein accumulation: inositol‐requiring enzyme 1 (Ire1)–X‐box binding protein‐1 (Xbp1), PKR‐like ER kinase (Perk)–activating transcription factor 4 (Atf4), and activating transcription factor 6 (Atf6) pathways (Hetz 2012). In the Ire1–Xbp1s pathway, ER stress stimulates the dimerization and subsequent trans‐autophosphorylation of Ire1 and thereby facilitates RNase activity of Ire1. Activated Ire1 splices a 26‐nucleotide intron from Xbp1 mRNA to produce the transcription factor Xbp1s (spliced Xbp1). Xbp1s stimulates the expression of a cluster of genes related to protein folding and quality control mechanisms (Hetz 2012).

Previous studies in model organisms have revealed alterations in ER stress‐response activity during aging (Martínez et al. 2017). In *C. elegans* , the ability to respond to pharmacological inducers of ER stress is lost with age (Ben‐Zvi et al. 2009), whereas in the *Drosophila* intestine, the Ire1‐Xbp1s pathway is activated during aging, which induces abnormal proliferation of aged intestinal cells (Wang et al. 2015). Several studies in mice have shown that the ER stress‐response is activated during aging (Martínez et al. 2017). Increased levels of Atf4 and Xbp1s were found in aged mouse osteocytes compared to young adults (Chalil et al. 2015). Similarly, adipose stromal cells in aged mice show elevated levels of Atf6 and phosphorylated Ire1 (Ghosh et al. 2015). These results suggest that ER stress and its response pathways are dysregulated with age and are associated with the progression of tissue aging. Changes in the ER stress‐response have been considered to be linked to aging progression, but it is still poorly understood how these changes affect the vertebrate aging process, in part because the age‐dependent alteration of the ER stress‐response has been examined only in limited organs and cells. To uncover this, analyzing the dynamics and roles of the ER stress‐response throughout the vertebrate lifespan would be effective. However, the long lifespan of conventional vertebrate models (approximately 3 years for mice and zebrafish) has hampered our understanding. To overcome this hurdle, we utilized the African turquoise killifish, *Nothobranchius furzeri* , which is an extremely short‐lived (several months) small vertebrate (Harel et al. 2016), to investigate the spatial dynamics and roles of ER stress‐response activity during the aging process.

## 2 Results

### 2.1 Age‐Dependent Changes of Ire1‐Xbp1s Activity in *N. furzeri*

**FIGURE 1:**
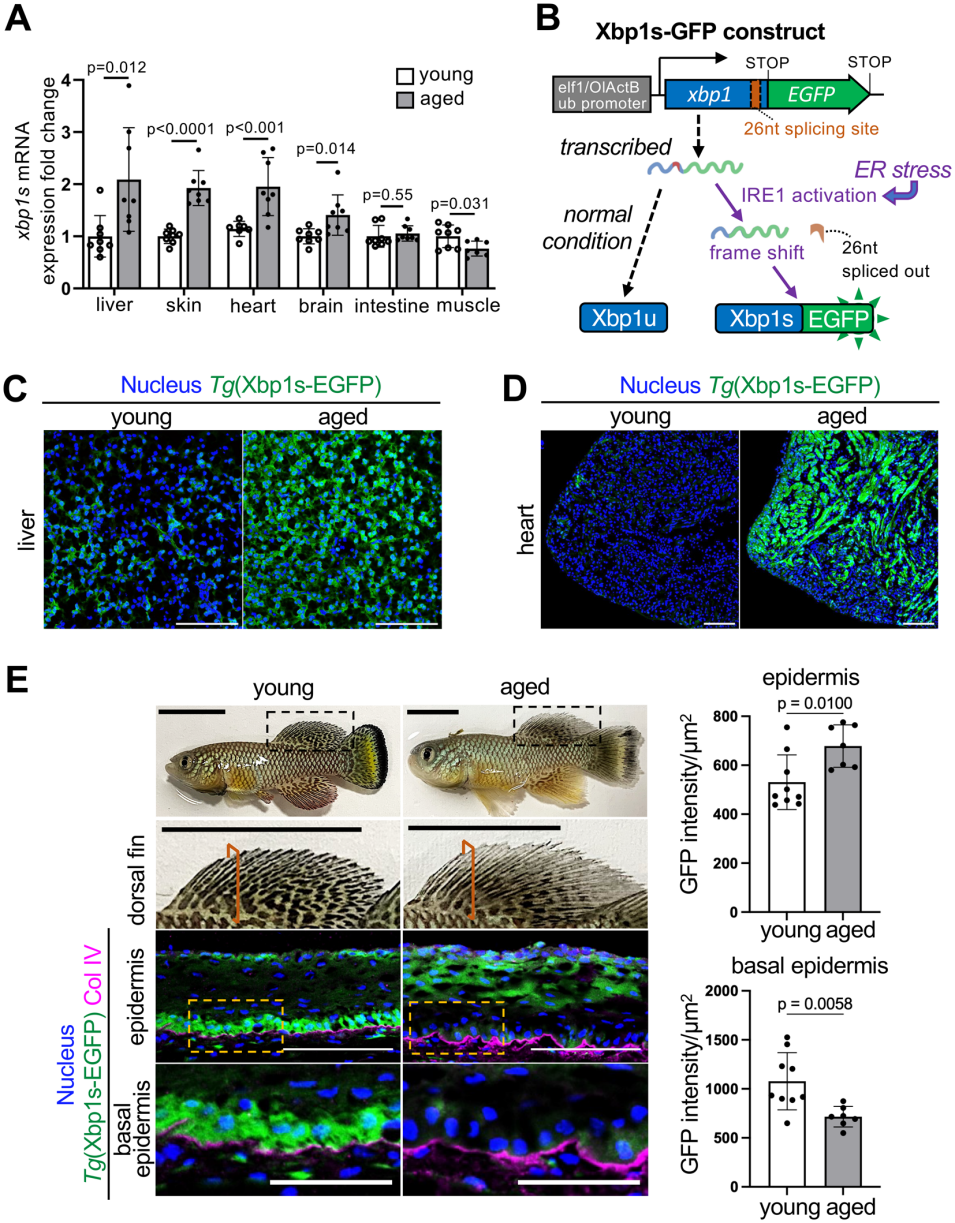
Age‐dependent activity change of the Ire1‐Xbp1s ER stress‐response pathway in *N. furzeri* . (A) *xbp1* spliced variant (*xbp1s*) mRNA levels in various tissues of young and aged *N. furzeri* shown as fold change relative to young. Values are mean ± s.d., *n* = 8; *p* = Student's *t*‐test. (B) Schematic illustration of ER stress‐response reporter construct. (C, D) Representative images of reporter activity in young and aged *N. furzeri* livers (C) and hearts (D) Scale bars, 100 μm. (E) Representative images of young (1.2 month) and aged *N. furzeri* (4.2 month) (top panels) and dorsal fin area (2nd row panels), reporter activity in young and aged *N. furzeri* fin epidermis (3rd row and bottom panels), and mean GFP intensity. Col IV (Collagen type IV) was used as a basement membrane marker. Black dashed line indicates dorsal fin areas shown in the 2nd row panels, red lines indicate sectioned area of dorsal fin shown in the 3rd and bottom row panels, and yellow dashed lines indicate basal epidermis areas shown in the bottom panels. Scale bars, 10 mm in dorsal fin, 100 μm in epidermis, and 50 μm in basal epidermis panels. Values are mean ± s.d., *n* = 9 for young and *n* = 7 for aged samples; *p* = Student's *t*‐test.

To examine changes in the ER stress‐response that occur during aging, we focused on the Ire1‐Xbp1s pathway, which is the most conserved and specific pathway of the ER stress‐response (Kohno 2010). We first confirmed that the 26 nucleotide intron of *N. furzeri xbp1* transcript was spliced out, similar to zebrafish, mouse, and human *xbp1* (Oikawa et al. 2012; Li et al. 2015) (Figure S1A) and that treatment with tunicamycin, an N‐glycosylation inhibitor that induces unfolded protein accumulation specific in the ER lumen (a pharmacological ER stress inducer) (Iwawaki et al. 2004), promoted *xbp1s* production in *N. furzeri* embryos (Figure S1B), suggesting that the ER stress‐response Ire1‐Xbp1s pathway works in *N. furzeri* . To evaluate age‐dependent changes in Ire1‐Xbp1s activity, we examined the expression levels of *xbp1s* mRNA in the liver, skin, heart, brain, intestine, and muscle of young (1.2 months) and aged (4.2 months) fish. Consistent with previous studies in mice (Xiong et al. 2014; Chen et al. 2023), *xbp1s* expression was upregulated in most aged tissues, including the liver and heart, compared to that in young tissues (Figure 1A). To further examine the spatiotemporal dynamics of ER stress‐response activity, we introduced the Xbp1s‐EGFP reporter (Iwawaki et al. 2004), which expresses a truncated Xbp1s‐EGFP fusion protein through endogenous Ire1‐dependent splicing in response to ER stress (Figure 1B), into *N. furzeri* via Tol2 transposase‐mediated transgenesis (Kawakami et al. 2004). We obtained a stable line carrying a single copy of the transgene by outcrossing 2 times with the wild‐type *N. furzeri* (Figure S1C). To test the specificity of the reporter, we treated the reporter‐transgenic embryos with tunicamycin and 4μ8C, an inhibitor of Ire1. As expected, tunicamycin activated the reporter expression, but co‐treatment with 4μ8C blocked this activation (Figure S1D,E), suggesting that the reporter specifically reflects the Ire1‐Xbp1s activity.

Next, we attempted to detect age‐dependent spatial changes in Ire1‐Xbp1s activity using the Xbp1s‐EGFP reporter in *N. furzeri* . Consistent with the qPCR results (Figure 1A), the number of GFP‐positive cells increased in the liver and heart with age (Figure 1C,D and Figure S2A,B). In aged livers, where *xbp1s* mRNA expression was upregulated the most compared to other tissues, unfolded protein aggregation and changes in ER shape and size, which are signs of ER stress, were also observed, indicating that ER stress may accumulate in the liver during aging (Figure S2C,D). In addition, the expression levels of ER stress‐response target genes encoding chaperone proteins (*hspa5* and *hsp90b1*), ER‐associated degradation machinery proteins (*edem1* and *derl1*), ER oxidase (*ero1*), and cell cycle arrest and apoptosis‐inducing transcription factor (*ddit3*) (Hetz 2012) were relatively higher in aged livers than in young fish (Figure S2E). These results suggested that chronic ER stress occurs in aged livers, consistent with previous observations in mouse livers (Gaspar et al. 2020; Lefebvre and Staels 2014).

### 2.2 Ire1‐Xbp1s Activity Drives Young Epidermal Cell Proliferation

**FIGURE 2:**
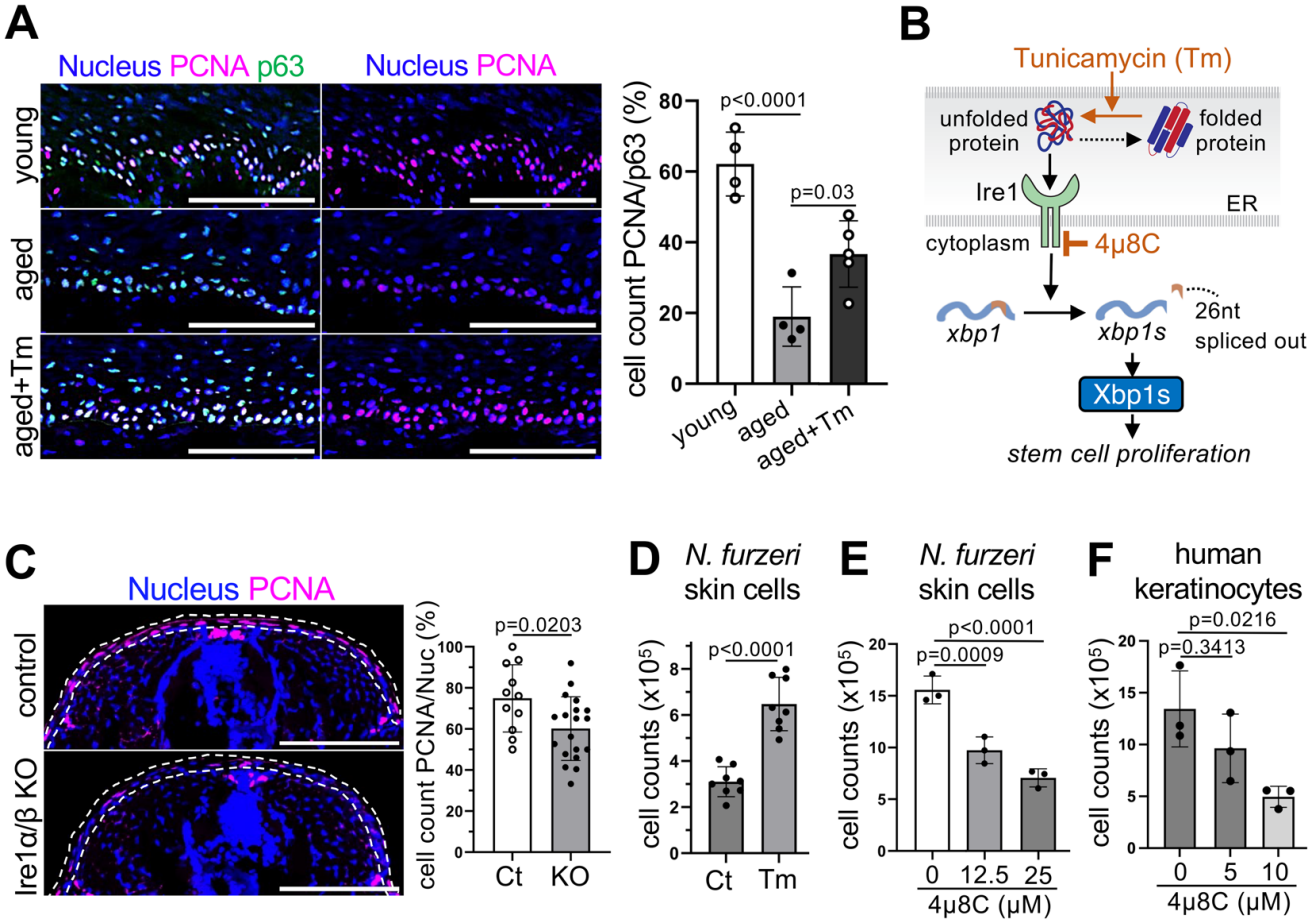
Ire1‐Xbp1s ER stress‐response activity is involved in epidermal cell proliferation. (A) Representative images and quantification of PCNA‐positive cells in young, aged, and tunicamycin‐treated aged (aged Tm) *N. furzeri* fins normalized to p63. Scale bars, 100 μm. Values are mean ± s.d., *n* = 4, 4, and 5; *p* = Dunnett's multiple comparisons of one‐way ANOVA of aged samples. (B) The schematic diagram of Ire1‐Xbp1 pathway and mechanism of tunicamycin (Tm) and 4μ8C. (C) Representative images and quantification of PCNA positive cells in dorsal epidermal layer of larva of control, and Ire1α (*ern1*) and Ire1β (*ern2*) double knock‐out. Scale bars, 100 μm. Values are mean ± s.d., *n* (slice) = 11, 19 and *N* (sample) = 6, 9; *p* = Student's *t*‐test. (D) Counts of tunicamycin (Tm)‐treated *N. furzeri* cells. Values are mean ± s.d., *n* = 8; *p* = Student's *t* test. (E, F) Ire1 activity drives the proliferation of *N. furzeri* skin culture cells (E) and human keratinocytes (F). Cells were treated with IRE1 inhibitor (4μ8C) and the number of cells were counted. Values are mean ± s.d., *n* = 3; *p* = Dunnett's multiple comparison of one‐way ANOVA of control.

Similar to the liver and heart, Xbp1s‐EGFP‐positive cells also increased in the epidermis during aging (Figure 1E). Consistently, FK1 (multi‐ubiquitin mAb)‐positive foci increased in aged epidermis (Figure S3A), suggesting an accumulation of unfolded protein in aged epidermis. In contrast, strong reporter activity was detected in the basal layer of the epidermis of young fish, which was significantly downregulated with age (Figure 1E). On the other hand, ubiquitinylated proteins' increase was not detected in the epidermal basal layer of both young and aged fish (Figure S3A), indicating that the strong activation of Ire1‐Xbp1s in the young epidermal basal layer may be independent of unfolded protein accumulation. Additionally, we observed a decrease in phosphorylated IRE1 at Ser‐724, which is an indicator of Ire1 activation (Hetz 2012), in the aged mouse epidermal basal layer (Figure S3B,C), suggesting that Ire1‐Xbp1s pathways also decline in mammalian epidermal basal layer during aging. Because the basal layer of the epidermis consists of epidermal stem cells and progenitors, we wondered whether Ire1‐Xbp1s activity or ER stress itself may be involved in the regulation of basal layer proliferation. Previous studies in humans and mice indicated that cell proliferation, including those in stem cells, decreases in aging epidermis (Liu et al. 2019; Giangreco et al. 2008; Inomata et al. 2009). Although keratinocyte stem cells and progenitors labeled with p63 (Pellegrini et al. 2001) were detected in both young and aged *N. furzeri* skin and did not change in number with age (Figure S4A), PCNA (proliferation marker)‐ and p63‐double positive cells decreased with age (Figure 2A), indicating that an age‐dependent decline in epidermal cell proliferation also occurs in *N. furzeri* . To test whether ER stress affected cell proliferation, aged fish were treated with tunicamycin. Transient treatment with tunicamycin increased the number of PCNA‐positive cells in the aged epidermal basal layer (Figure 2A,B). We also confirmed that tunicamycin induced the expression of endogenous mRNA levels of *xbp1s*, ER stress‐response‐target genes (*hspa5* and *hsp90b1*), and Xbp1s‐EGFP reporter activity in aged *N. furzeri* skin (Figure S4B,C), whereas levels of ubiquitinylated protein, cell death, and cellular adherens junction showed no significant changes (Figure S4D–F), suggesting that transient activation of ER stress response did not induce further damages to epidermal cells. Notably, tunicamycin treatment also showed a tendency of increased cell proliferation in the young epidermal basal layer based on PCNA‐positive cell count (Figure S4G). Together, these results suggest that the high ER stress‐response in the epidermal basal layer drives cell proliferation, and forced activation of ER stress can rejuvenate basal layer activity in aged epidermis.

To further confirm that Ire1‐Xbp1s activity is involved in epidermal cell proliferation, we knocked out genes encoding Ire1 receptors using the triple‐target CRISPR‐Cas9 method, which enables single gene disruption to efficiently produce whole‐body biallelic knockouts and examine gene function in the F0 generation (Oginuma et al. 2022). A previous study in medaka ( *Oryzias latipes* ) suggested that the loss of both Ire1α and Ire1β is required to suppress *xbp1* splicing (Ishikawa et al. 2017). Therefore, we knocked out both Ire1α and Ire1β (encoded in *ern1* and *ern2* genes, respectively) in *N. furzeri* (Figure S5A) and confirmed that the expression of *ern1* and *ern2* was suppressed in the KO embryos (Figure S5B). As expected, Ire1α/β KO reduced PCNA‐positive cells in young fish (Figure 2C), suggesting that Ire1 contributes to epidermal cell proliferation. To further confirm this hypothesis, we used young *N. furzeri* skin‐derived epidermal culture cells expressing the progenitor cell marker p63 (Figure S6A). Consistent with in vivo results, tunicamycin treatment increased proliferative capability in *N. furzeri* skin culture cells (Figure 2D and Figure S6B), while treatment with a specific Ire1 chemical inhibitor 4 μ8C decreased it in a dose‐dependent manner (Figure 2E and Figure S6C), without inducing cell death (Figure S6D). Collectively, these data suggest that Ire1‐Xbp1s activity is involved in cell proliferation. In addition, 4μ8C treatment also reduced the proliferation of human keratinocyte PHK‐160b cells (Figure 2F), suggesting that the Ire1‐Xbp1s pathway‐mediated epidermal cell proliferation is conserved in humans.

Interestingly, strong activation of the Xbp1s‐GFP reporter was also detected in the brain mesencephalic proliferation zone (Fan et al. 2001) and proliferative intestinal crypts (Barker et al. 2007) of young *N. furzeri* (Figure S7A,B), suggesting that Ire1‐Xbp1s activity may contribute to cell proliferation in a variety of young tissues, including the brain and intestine.

### 2.3 ER Stress Rejuvenates Transcriptomic Patterns of Aged Epidermal Cell Proliferation

**FIGURE 3:**
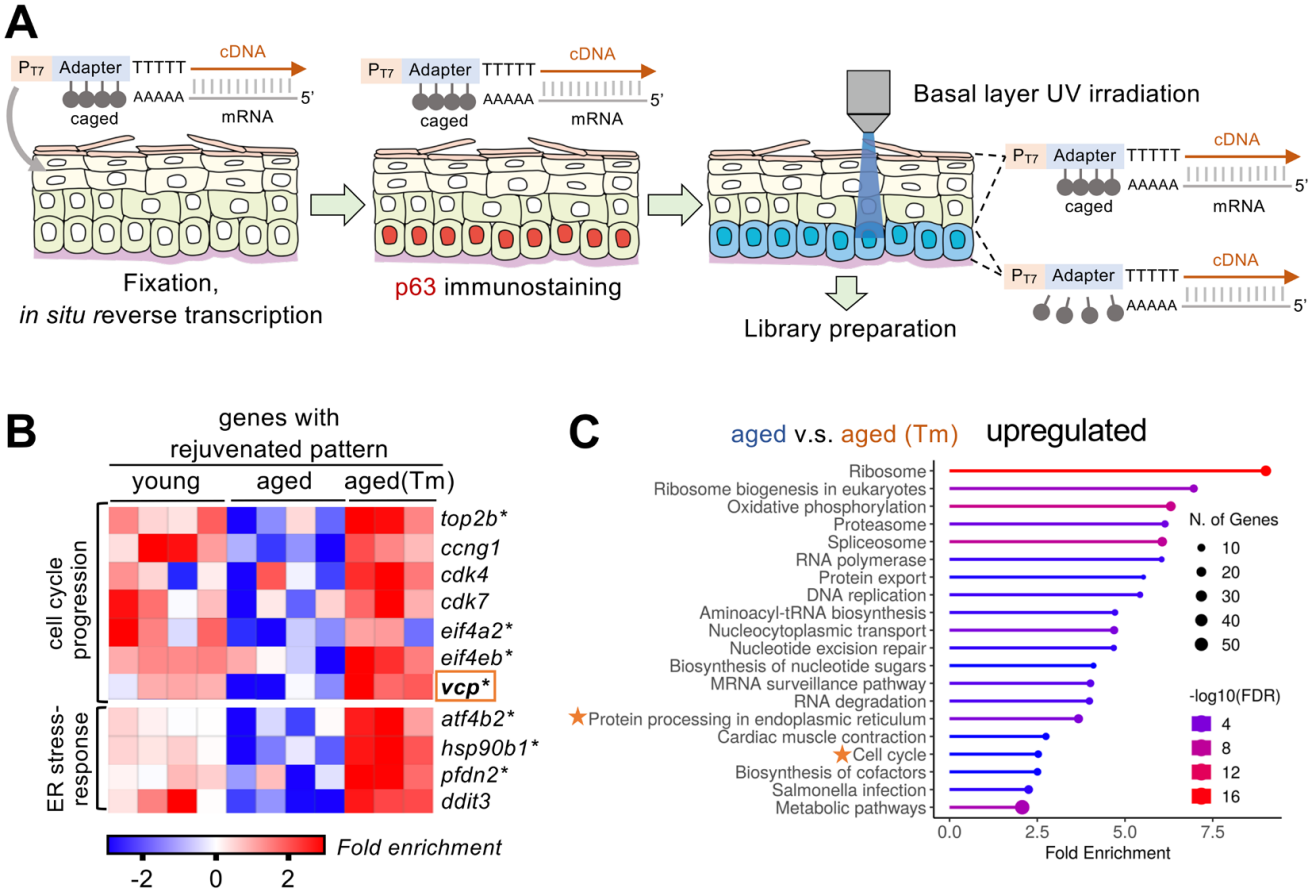
Spatial transcriptomics PIC reveals transcriptome pattern of aged epidermal basal layer is partially rejuvenated after ER stress forced activation. (A) Schematic illustration of photo‐isolation chemistry (PIC) in the epidermal basal layer. (B) Differentially expressed genes (DEG) of genes whose expressions were rejuvenated after tunicamycin treatment and DEG of ER stress‐response and protein folding‐related genes. *n* = 4, 4, and 3 for young, aged, and aged (Tm), respectively. Asterisks (*) indicate genes with FDR < 0.1 compared with aged (Tm). (C) GO term analysis of genes that were upregulated in the basal layer of aged Tm compared to aged.

To elucidate the mechanism by which the Ire1‐Xbp1s pathway is regulated and controls epidermal cell proliferation, we conducted a spatial transcriptomic analysis of young, aged, and tunicamycin‐treated aged (aged (Tm)) epidermal p63‐positive cells using photo‐isolation chemistry (PIC) (Honda et al. 2021). PIC uses a photosensitive probe that enables the determination of gene expression profiles specifically from photo‐irradiated regions of interest (Figure 3A and Figure S8A). PCA analysis from PC1 and PC2 showed a stark contrast between aged (Tm) and young and aged transcriptome patterns, which is caused by an elevated stress response as expected (Figure S8B). However, PCA analysis from PC2 and PC3 showed that the transcriptomic patterns of certain groups of genes were clearly different between young and old epidermal basal layers, and tunicamycin treatment partially restored the aged patterns to young ones (Figure S8B). These results indicate that forced activation of ER stress can partially rejuvenate the transcriptomic patterns of the aged epidermal basal layer. This rejuvenated group included genes related to cell cycle progression (*top2*, *cdk4*, *cdk7*, and *eif4*) (Whitfield et al. 2006) and ER stress response (*atf4*, *hsp90b1*, *pfdn2*, and *ddit3*), which were downregulated with age but upregulated in aged (Tm) samples (Figure 3B and Figure S8C). Pathway analyses also indicated that tunicamycin treatment activated the expression of genes related to protein processing in the ER and the cell cycle in the aged basal layer (Figure 3C). These changes in expression patterns are consistent with our observation that Ire1‐Xbp1s activity and epidermal cell proliferation are downregulated with aging, whereas age‐dependent downregulation is reversed by tunicamycin treatment. Notably, our PIC analyses also demonstrated that age‐dependent downregulation of *hnrnpu* and upregulation of the map‐Semaphorin (*plxna2*, *sema6dl*, and *sema3ab*) and Notch signaling (*dll4*)‐related genes were reversed by forced ER stress activation (Figure S8D). Consistently, the downregulation of *hnrnpu* in mice leads to epidermal thinning, skin fragility, and dysregulation of differentiation, while the Plexin‐Semaphorin signaling and activation of the Notch signaling negatively regulate epidermal cell proliferation (Hong et al. 2024; Jiang et al. 2021). These results show that the age‐dependent reduction of *hnrnpu* expression and activation of the Plexin‐Semaphorin and Notch signaling would promote aging in *N. furzeri* epidermis. Taken together, our PIC analyses revealed the age‐dependent transcriptomic pattern changes in the *N. furzeri* epidermal basal layer and that ER stress rejuvenates various activities of the aged epidermal basal layer.

### 2.4 Ire1‐Xbp1s Promotes Epidermal Cell Proliferation Through Vcp

**FIGURE 4:**
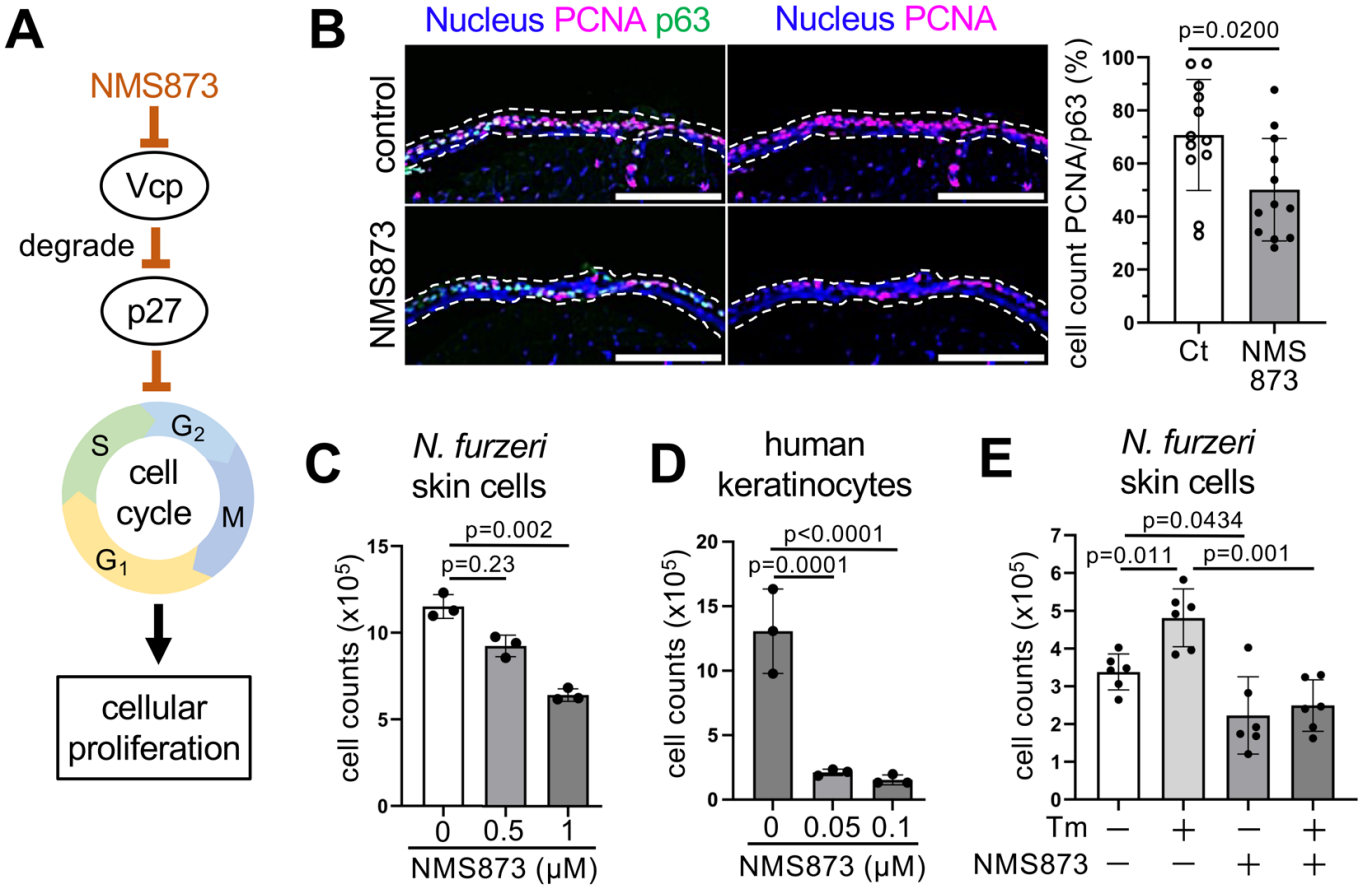
VCP is required for Ire1‐Xbp1s‐dependent epidermal cell proliferation. (A) Mechanism of Vcp‐mediated cell cycle regulation and Vcp inhibition (NMS873). (B) Representative images and quantification of PCNA positive cells of the control and Vcp inhibitor (NMS873) were used to treat the dorsal epidermal layer of 2‐week old *N. furzeri* larvae normalized to p63. Scale bars, 100 μm. Values are mean ± s.d., *n* = 12; *p* = Students' *t*‐test. (C, D) VCP activity is required for the proliferation of *N. furzeri* skin culture cells (C) and human keratinocytes (D). Cells were treated with NMS873 and the number of cells was counted. Values are the means ± s.d., *n* = 3; *p* = Dunnett's multiple comparisons of one‐way ANOVA of control. (E) Cells were treated with tunicamycin (5 μg/mL) or NMS873 (1 μM) or both, and the number of cells was counted. Values are the means ± s.d., *n* = 6; *p* = Dunnett's multiple comparisons of one‐way ANOVA of control.

Among the rejuvenated group genes, we focused on valosin‐containing protein (Vcp), an evolutionarily conserved ATPase, as a candidate mediator of Ire1‐Xbp1s‐dependent proliferation because Vcp positively regulates the G1 to S phase transition in cell cycle progression by targeting the nuclear export and degradation of the CDK inhibitor p27 and is also involved in the regulation of ER‐associated degradation (ERAD), which is a downstream process of the ER‐stress response (Figure 3B) (Meyer et al. 2012). Consistently, the human age‐related gene expression database voyAGEr (Schneider et al. 2024) shows that *vcp* and several ER stress‐response‐related genes were downregulated with age in human suprapubic skin (Figure S8E). To examine whether Vcp activity is involved in epidermal cell proliferation, we treated young fish with NMS‐873, a specific chemical inhibitor of Vcp, and found that NMS‐873 treatment reduced the number of PCNA‐positive epidermal cells (Figure 4A,B). In addition, NMS‐873 treatment also decreased the proliferation of *N. furzeri* skin culture cells and human keratinocytes in a concentration‐dependent manner (Figure 4C,D). Furthermore, tunicamycin‐induced *N. furzeri* skin culture cell proliferation was significantly attenuated when co‐treated with NMS‐873 (Figure 4E), suggesting that Ire1‐Xbp1s drives epidermal cell proliferation in a Vcp activity‐dependent manner.

### 2.5 Glucose Activates Ire1‐Xbp1s in Young Epidermal Basal Layer

**FIGURE 5:**
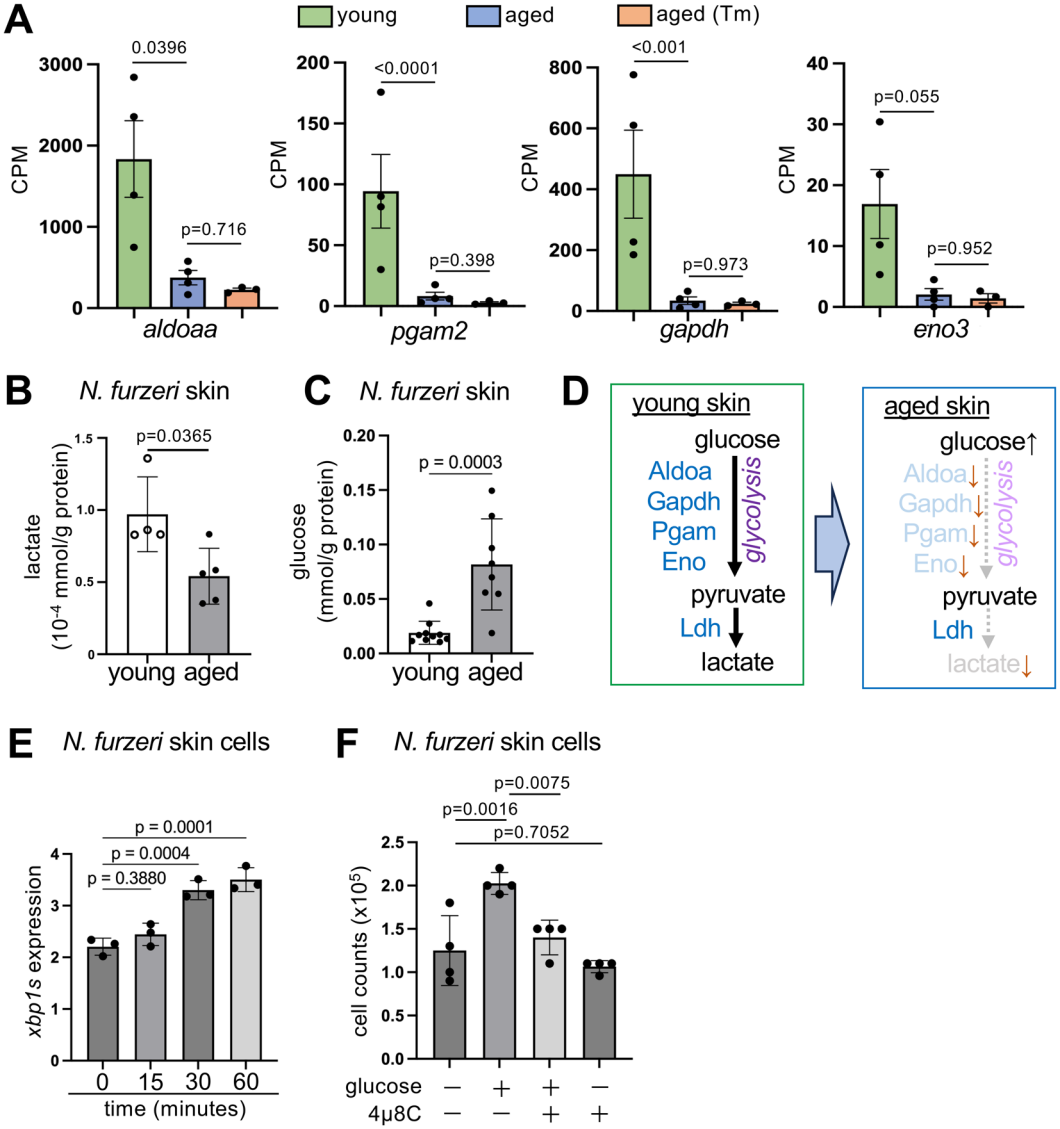
Glucose activates Ire1‐Xbp1s pathway in young epidermal cells. (A) Gene expressions in Count per Million (CPM) that were downregulated in aged and aged Tm basal epidermal basal layers compared to young Tm. Values are mean ± s.d., *n* = 4, 4, and 3 for young, aged, and aged (Tm), respectively; *p* = adjusted *p* value. (B, C) Lactic acid (B) and glucose (C) assays of young and aged fin samples. Values are mean ± s.d., *n* = 4,5; *p* = Student's *t*‐test. (D) Schematic diagram of glucose metabolism in young and aged epidermal stem cells. (E) *xbp1* spliced variant (*xbp1s*) mRNA levels at 0, 15, 30, and 60 min after glucose treatment (4.5 g/L). Values are mean ± s.d., *n* = 3; *p* = Dunnett's multiple comparisons of one‐way ANOVA to 0 min. (F) Cells were treated with glucose (4.5 g/L), 4μ8C (25 μM), or both, and the number of cells was counted. Values are the means ± s.d., *n* = 6; *p* = Dunnett's multiple comparisons of one‐way ANOVA to non‐treated control or glucose only‐treated samples.

The PIC data also indicated that genes related to glycolysis, including *aldoaa*, *gapdh*, *pgam2*, and *eno3*, were downregulated with age (Figure 5A and Figure S8F). In addition, lactate, a product of glycolysis, decreased significantly in aged skin, accompanied by an accumulation of glucose (Figure 5B–D), suggesting a reduced ability to metabolize glucose and an overall decline in glycolysis in aged epidermal cells (Jackson and Finley 2024). A previous study in pancreatic β‐cells showed that Ire1‐Xbp1s is activated when treated with glucose, the precursor of glycolysis (Sharma et al. 2015). Therefore, we investigated whether glucose could induce Ire1‐Xbp1s activity via glycolysis. Consistent with this, we found that glucose treatment induced Ire1‐Xbp1s activity (Figure 5E) and proliferation in *N. furzeri* skin cells, whereas inhibition of Ire1 using 4μ8C suppressed the glucose‐dependent proliferative effect (Figure 5F). These findings suggest that the Ire1‐Xbp1s pathway is activated by glucose and that the downregulation of glucose metabolism during aging may consequently reduce Ire1‐Xbp1s activity and cellular proliferation in the epidermal basal layer.

## 3 Discussion

**FIGURE 6:**
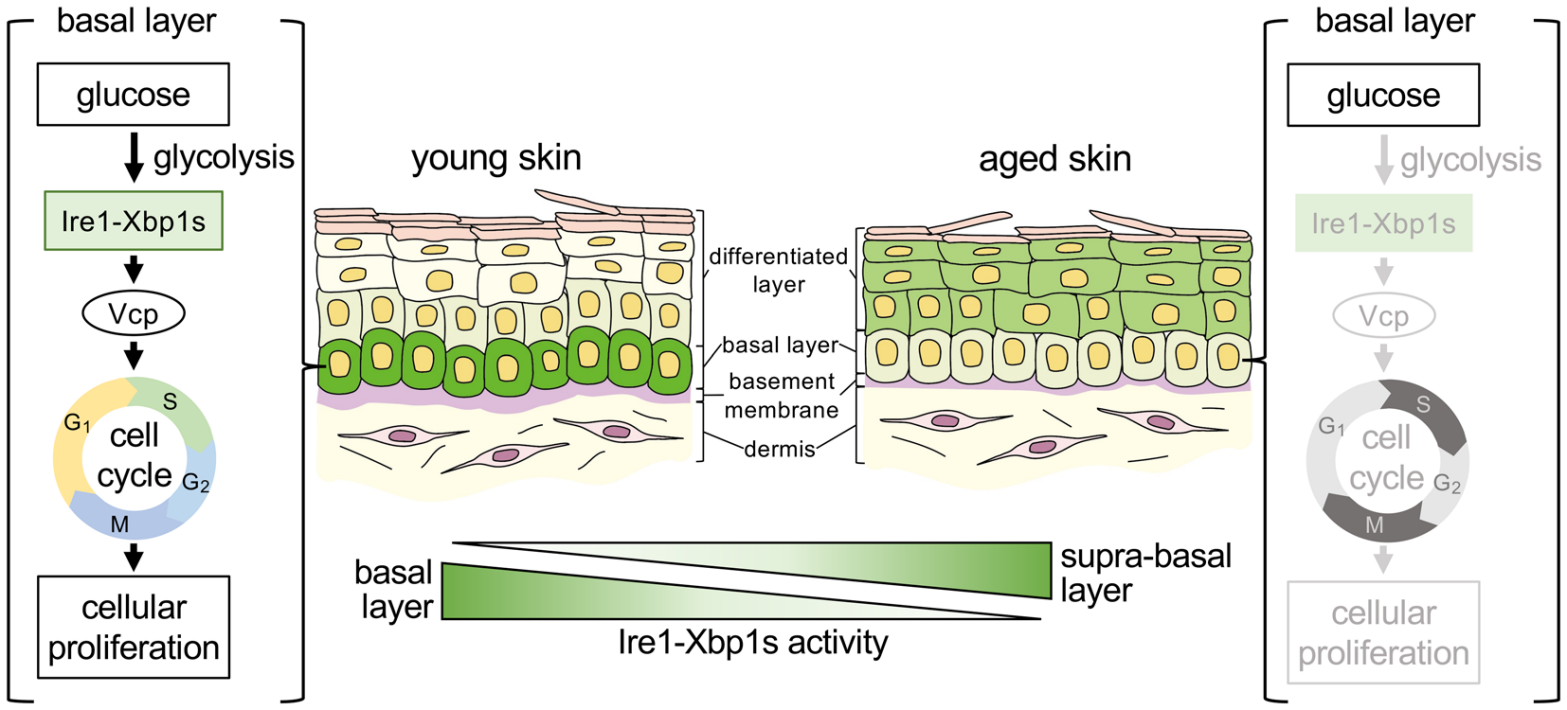
Schematic of the mechanism of Ire1‐Xbp1s‐mediated epidermal cell proliferation and age‐dependent decline. In young epidermal basal layer, the Ire1‐Xbp1s endoplasmic reticulum (ER) stress‐response pathway maintains cell proliferation by activating the cell cycle regulator Vcp, whereas the age‐dependent decline in glucose metabolism reduces Ire1‐Xbp1s activity, consequently downregulating cell proliferation. On the other hand, in supra‐basal layer, the Ire1‐Xbp1s pathway is activated with age.

We showed the aging‐associated dynamics and an unexpected role of the ER stress‐response pathway in regulating epidermal tissue homeostasis and aging (Figure 6) using *N. furzeri* and human cell cultures. Previous studies have reported that the age‐dependent unfolded protein accumulation‐mediated ER stress and sustained activation of the ER stress‐response are linked to various aging‐associated diseases (Chen et al. 2023; Boslem et al. 2023; Lefebvre and Staels 2014). Consistent with these observations, our qPCR and reporter analyses revealed that the ER stress‐response Ire1‐Xbp1s pathway is upregulated in various tissues of *N. furzeri*, including the liver and heart, with age. Surprisingly, Ire1‐Xbp1s was highly activated in the young *N. furzeri* epidermal basal layer and downregulated during aging. In young skin, the Ire1‐Xbp1s pathway maintains cell proliferation by activating the cell cycle regulator, Vcp. The activation of Ire1‐Xbp1s in the epidermal basal layer is driven by glucose metabolism, which also declines during aging. The glucose‐Ire1‐Xbp1s‐VCP axis also positively regulates the proliferation of human skin cell cultures. These results indicate that the ER stress‐response may control epidermal cell aging by sustaining its proliferative activity.

ER stress has been regarded as detrimental; however, its beneficial effects are not well understood. In addition, studies on Ire1‐Xbp1s have focused on its role as an adaptive mechanism against misfolded protein aggregation stress; however, its other functions remain unclear. Although recent studies show that the Ire1‐Xbp1s pathway can promote proliferation of cancer cells in vitro (Ji et al. 2019) and satellite cells in mouse injured muscle (Roy et al. 2021), and pharmacological activation of ER stress stimulates β cell proliferation in vitro, the mechanism connecting ER stress response and cell proliferation remains unclear (Sharma et al. 2015). Our reporter and spatial transcriptome analyses revealed a previously unrecognized role for ER stress signaling in the positive regulation of epidermal cell proliferation through Ire1‐Xbp1s to maintain skin homeostasis. We also demonstrated that age‐dependent downregulation of Ire1‐Xbp1s activity correlates with aging phenotype in the epidermal basal layer, whereas previous studies proposed that age‐dependent upregulation of the ER stress response is a driver of aging (Martínez et al. 2017; Wang et al. 2015; Ghosh et al. 2015).

Furthermore, we successfully reactivated aged skin epidermal cells by pharmacologically activating ER stress. It is well understood that organismal aging is affected by surrounding environmental stimuli, including cellular stress (Kourtis and Tavernarakis 2011). Previous studies on *C. elegans* have shown that oxidative stress during the larval stage may induce a hormesis state that enhances stress resistance and longevity (Bazopoulou et al. 2019). Similarly, the ER stress‐response was recently shown to regulate cellular responses in human hematopoietic stem cells, where, under ER stress, human hematopoietic HSCs upregulated the pro‐survival Ire1‐Xbp1s branch to survive (van Galen et al. 2014). This indicates the possibility that moderate ER stress signaling and specific activation of the Ire1‐Xbp1s pathway could be beneficial for maintaining tissue homeostasis. Additionally, our observation that cytoplasmic ubiquitination was not detected in young epidermal basal layers suggests that strong Ire1‐Xbp1s activity in the young epidermal basal layer may be independent of unfolded protein; however, it is also important to note that activation of Ire1‐Xbp1s, to some extent, may reduce the accumulation of unfolded protein.

Interestingly, the young brain mesencephalic region also shows strong Ire1‐Xbp1s activity, which declines with age, suggesting that Ire1‐Xbp1s activity may contribute to the maintenance of youthfulness in the epidermal basal layer, and this decline drives aging not only in the skin but also in other tissues. In contrast, the intestinal crypt Ire1‐Xbp1s activity tended to increase with age. The increase in Ire1‐Xbp1s activity in aged *N. furzeri* intestinal crypts might indicate abnormal cell proliferation, similar to findings in *Drosophila* intestines, where the Ire1‐Xbp1s pathway upregulation during aging causes abnormal intestinal cell proliferation (Wang et al. 2015). Further investigation of various tissues is necessary to better understand the roles and significance of the Ire1‐Xbp1s pathway‐mediated control of cell proliferation in tissue homeostasis.

The application of PIC, a novel spatial transcriptomics method, enabled the analysis of transcriptomes specific to the epidermal basal layer of *N. furzeri* and identify glucose metabolism and Vcp as upstream and downstream mediators of the Ire1‐Xbp1s pathway, respectively, in epidermal cell proliferation. Vcp has been well studied as a positive regulator of the cell cycle and cancer progression (Meyer et al. 2012); however, its roles and regulation in tissue homeostasis and cell proliferation are poorly understood. This study revealed that Vcp activity is essential for Ire1‐Xbp1s‐mediated *N. furzeri* epidermal cell proliferation. Notably, Vcp is also a known mediator of protein degradation during ER‐associated degradation (ERAD) which is activated during the ER stress‐response (Wolf and Stolz 2012). In young skin epidermal basal layer, it is possible that Vcp plays a role not only in cell cycle control but also in protection from proteotoxic insults downstream of the Ire1‐Xbp1s ER stress‐response.

Several studies have reported that reduced glucose metabolism correlates with an aging phenotype. For example, in *Drosophila*, aging leads to a reduction in the expression of glycolytic genes in the muscle and brain, and the forced expression of glycolytic genes extends a healthy lifespan (Ma et al. 2018; Oka et al. 2021). Glycolysis is also impaired in aged human brain and aged mouse endothelial cells (Kiesworo et al. 2024). In this study, we showed that glucose promotes *N. furzeri* epidermal cell proliferation by activating the Ire1‐Xbp1s pathway and that age‐dependent reduction of glucose metabolism may downregulate the Ire1‐Xbp1s pathway to promote *N. furzeri* epidermal cell aging. Even though glucose‐dependent activation of Ire1‐Xbp1s was also observed in pancreatic β‐cells (Sharma et al. 2015), it remains unclear how glucose stimulates Ire1‐Xbp1s via its metabolism. A study on hepatoma cells suggested that the tricarboxylic acid (TCA) cycle, a metabolic pathway downstream of glucose, could stimulate ER stress through *Idh2*‐mediated mitochondrial NADPH production and glutathione redox (Gansemer et al. 2020), indicating the possibility that glucose might activate Ire1‐Xbp1s activity through this TCA cycle‐mediated mechanism in *N. furzeri* skin. A previous study also showed that inhibiting lactate production by knocking out *Ldh*, an enzyme that produces lactate, suppressed stem cell proliferation in hair follicles, suggesting that increased production of lactate or other associated metabolites from glycolysis is essential for stem cell activation (Flores et al. 2017). Thus, it might also be possible that lactate or other *Ldh*‐associated metabolites are involved in the ER stress‐mediated epidermal cell activation (Sun et al. 2024). Further elucidation of the relationship between cell metabolism and the ER stress response is necessary to uncover the cause of declining proliferation in epidermal cells.

Importantly, the human public data also showed an age‐dependent reduction in the expression of ER stress‐response‐related genes, Vcp, and glycolysis‐related genes in human skin, similar to *N. furzeri* (Figure S8E) (Schneider et al. 2024). These findings suggest that the glucose‐Ire1‐Xbp1s‐Vcp axis may contribute to the maintenance and aging of young human epidermal cells. Further understanding of the roles and regulation of Ire1‐Xbp1s activity in mammalian and human epidermal stem cells may lead to the discovery of promising therapeutic strategies for promoting healthy skin aging in humans.

## 4 Materials and Methods

### 4.1 Annual African Turquoise Killifish *N. furzeri* Strain, Husbandry, and Maintenance

The GRZ (GRZ‐AD) strain of *N. furzeri* was donated by A. Antebi (Max Planck Institute for Biology of Aging). Fish were maintained at 26.5°C, 0.7 conductivity on a 12‐h light–dark cycle in the fish breeding system (Meito, Nagoya, Japan). Click or tap here to enter text. Newly collected embryos were disinfected, washed, and stored in egg water (0.01% methylene blue in 3% sea salt) at 28°C, and moved to sterilized moist coconut peats at 7 days post fertilization (dpf) at 28°C. Around 4 to 5 weeks post‐fertilization, embryos were hatched in cold 700 mg/L humic acid (Cat no. 53680, Sigma‐Aldrich, St. Louis, MO, USA) in a 4 L tank, and then raised at 3 fish per 1.4 L tanks from 3 weeks old and 1 fish per 1.4 L tanks from 4 weeks old. The fish were fed freshly hatched brine shrimp (Brine Shrimp Eggs, Artemia Cyst, A&A Marine LLC., Kyoto, Japan) twice a day from Monday to Saturday and once a day on Sunday. From the age of 2 weeks, the fish were fed bloodworms (Clean Bloodworm, Kyorin Co. Ltd., Himeji, Japan) once a day. The study protocol was approved by the Institutional Animal Care and Use Committee of Osaka University (RIMD Permit#R02‐04). This study was conducted in accordance with the ARRIVE guidelines.

### 4.2 Mouse Strain, Husbandry, and Maintenance

Young and aged C57BL/6N mice were purchased from Sankyo Lab Service (Tokyo, Japan). All mice were maintained under specific pathogen‐free conditions with a 12‐h light/dark cycle and were fed a normal diet with ad libitum access to water. All experiments were conducted in accordance with the Guidelines for the Care and Use of Laboratory Animals and were approved by the Institutional Animal Care and Use Committees of The University of Tokyo.

### 4.3 Cell Lines

*N. furzeri* cell line was established as previously described (Graf et al. 2013). Skin and fins were collected from 1.5‐month‐old male *N. furzeri* and submerged in ice‐cold 0.16% NaClO_4_ in PBS and then washed in ice‐cold phosphate‐buffered saline (PBS), followed by shaking in DMEM containing collagenase for 2 h at RT. Cells were collected using a cell strainer and grown in DMEM (Cat no. 08458, Nacalai Tesque Inc.) supplemented with 10% FBS (Cat no. SFBU30, Equitech‐Bio Inc., Kerrville, TX, USA), amphotericin (Cat no. 1397‐89‐3, Sigma‐Aldrich), penicillin/streptomycin (Cat no. 09367‐34, Nacalai Tesque Inc.), gentamicin (Cat no. 078‐06061, FUJIFILM Wako Pure Chemical Co., Osaka, Japan), MEM‐NEAA (Cat no. 06344‐56, Nacalai Tesque Inc.), 2–mercaptoethanol (Cat no. 21438‐82, Nacalai Tesque Inc.), and FGF (Cat no. 450‐33A, Thermo Fisher Scientific) at 28°C. PHK16‐0b were grown in HuMedia‐KG2 supplemented with insulin, hEGF, hydrocortisone, gentamycin, amphotericin B, and BPE (Cat no. KK‐6150, Kurabo Industries Ltd., Osaka, Japan) at 37°C.

### 4.4 Genotyping and Genomic DNA Extraction From Scales and Fin Clips

Adult fish were genotyped using specific primers for either the GRZ or *Tg* line to confirm the identity of the strain. Around 5 sheets of scales were taken using forceps and incubated in 50 μL sodium hydroxide 50 mM (Cat no. 31511, Nacalai Tesque Inc.) at 95°C for 20 min, followed by 5 μL Tris–HCl pH 5.5. Samples were amplified using Ex Taq DNA polymerase (Cat. RR001B Takara Bio Inc., Kusatsu, Japan). Tail cutting was performed by clipping the caudal fin of 1‐month‐old fish. Fish were anesthetized by submerging them in 0.04% tricaine in system water. The clipped fin was incubated in lysis buffer containing 10 mM Tris–HCl pH 8.0 (Cat no. 35435‐11, Nacalai Tesque Inc.), 10 mM EDTA (Cat no. 15105‐22, Nacalai Tesque Inc.), 200 mM NaCl (Cat no. 31319‐45, Nacalai Tesque Inc.), and 0.5% SDS in ddH_2_O added with 40 μL/mL Proteinase K at 55°C overnight, followed by heat inactivation at 100°C for 10 min. Genomic DNA was extracted using phenol‐chloroform‐IAA and diluted in a Tris‐EDTA buffer.

### 4.5 Confirmation of *xbp1* Endogenous Splicing in *N. furzeri* Line

*N. furzeri* embryos around 7 d post fertilization (dpf) were treated with 5 μg/mL Tunicamycin (Cat no. 35638, Nacalai Tesque Inc.) in dimethyl sulfoxide (DMSO) (Cat no. 13408‐64, Nacalai Tesque Inc.) diluted in egg water for 24 h. RNA from embryos was extracted using TRIzol (Cat no. 15596018, Invitrogen, Waltham, Massachusetts, USA) followed by purification using RNA Clean and Concentrator‐25 (Cat no. R1017, Zymo Research, CA, USA). cDNA was synthesized using Transcriptor High Fidelity cDNA Synthesis Kit (Cat no. 05091284001 Roche diagnostics GmbH, Mannheim, Germany) and further amplified using PrimeSTAR Max DNA Polymerase (Cat no. R405A, Takara Bio Inc.). Poly‐A end was added by adding Ex Taq followed by incubation at 72°C for 20 min. Amplified products were run in a 3% agarose gel. Resulting bands were extracted separately using Nucleospin Gel and PCR Clean‐Up (Cat no. 740609, Macherey‐Nagel Inc., Allenton, PA, USA). Each fragment was cloned and sequenced using Sanger sequencing (Eurofin Genomics, Japan).

### 4.6 Generation of Transgenic Reporter *N. furzeri* Line Tg(Xbp1s‐GFP) Construct

*N. furzeri xbp1* fragment was synthesized using specific primers spanning bases 1219–1534, according to the NCBI database NC_029659.1 reference Nfu_20140520 primary assembly. Fragments were synthesized and extracted from 14 dpf embryo RNA, as previously described, and inserted into the pCS2P + Flag (#16331; Addgene) gifted by Peter Klein. Constructs with flag‐tagged fragments were inserted into the pT2‐Olactb‐Elf1 plasmid (Yoshinari et al. 2012).

### 4.7 Microinjection in *N. furzeri*

One‐cell stage embryos (bulging cell on top of the yolk) were collected by placing males and females in 4 L tanks with sand trays to breed for 2 h. Embryos were collected, rinsed, and positioned in the injection plate (3% agarose mold of 0.9 cm wide lanes). Microinjections were performed using the Narishige IM‐400 instrument. Injection needles were prepared using a 1 mm glass capillary G1 (Narishige Scientific Instrument Lab., Tokyo, Japan) pulled using a micropipette puller (Cat no. PC‐100, Narishige Scientific Instrument Lab.). The injection mix was loaded into the needles by back‐loading and injected into the bulging part of the embryos. Injection volume was set to be around 5 pL or around 20%–50% of the injected cell volume. Post‐injected embryos were rinsed and stored as described previously.

### 4.8 Genomic Southern Blot Analysis

Genomic DNA from *N. furzeri* tails was extracted as previously described and digested using EcoRI overnight. Southern blot hybridization was performed using a digoxigenin (DIG)‐labeled probe in accordance with DIG‐Easy Hybridization (Cat no. 11603558001, Roche diagnostics GmbH) standard method and detected by chemiluminescence using CDP Star (Cat no. 160081‐62‐9, Roche Diagnostics).

### 4.9 RNA Extraction and Quantitative Real‐Time Polymerase Chain Reaction (qRT‐PCR)

RNA from cells or tissues was extracted and cDNA was synthesized from 500 ng RNA as described above. Around 50 ng cDNA was used as a template for qRT‐PCR using THUNDERBIRD SYBR qPCR Mix (Cat no. QPS‐201, Toyobo Co. Ltd.). Gene expression was measured and analyzed using the Stratagene Mx3000P qPCR system (Agilent Technologies, Santa Clara, CA, USA) or CFX‐Duet (Bio‐Rad, Hercules, CA, USA), and normalized using *tbp* expression with the primers listed in Table S1.

### 4.10 Confirmation of Tg (Xbp1s‐GFP)

The Tg (Xbp1s‐GFP) embryos were collected as described above. 8 dpf embryos were treated with 0.05% DMSO or 5 μg/mL Tunicamycin or 5 μg/mL Tunicamycin and 2.5 mM 4μ8C (Cat no. S7272, Selleck Chemicals, Houston, TX, USA) for 2 h, with a medium change on the second day.

### 4.11 *N. furzeri* Tissue Sectioning and Immunochemistry

Fish were sacrificed as described above. Tissues were then collected and washed with cold PBS and fixed in 4% PFA in PBS (Cat no. 26126‐25, Nacalai Tesque Inc.) overnight at 4°C. Fixed tissues were washed with PBS 3 times for 15 min, followed by immersion in 10%, 20%, and 30% sucrose until tissue was fully submerged. Tissues were embedded with O.C.T. compound (Cat no. 4583, Tissue T‐Tek, Sakura Finetek Japan Co. Ltd., Tokyo, Japan) and flash frozen in liquid nitrogen, then stored at −80°C. Cryosectioning was then performed using HM525 NX cryostat (Thermo Fisher Scientific) at −25°C to −30°C. Tissue 8 μm sections were cut and transferred to adhesive slide glass (Matsunami Glass Ind. Ltd., Kishiwada, Japan), air‐dried at RT for 1 h, and stored at −80°C. Frozen sections were air‐dried at RT for 1 h followed by antigen retrieval using Dako target retrieval solution, citrate pH 6 (S236984‐2, Agilent Technologies Inc., Santa Clara, CA, USA) at 105°C for 15 min in autoclave, cooled to RT (for PCNA staining only), and then rinsed with 0.01% Triton in PBS (PBST 0.01%) for 10 min thrice, followed by blocking with 10% FBS in PBST 0.03% for 1 h at RT. Sections were then incubated with primary antibody anti‐GFP (13,970, Abcam, Cambridge, UK, 1:1000), or anti‐collagen type IV (ab6586, Abcam, 1:300), or anti‐multi‐ubiquitin mAb, clone FK1 (DO71‐3, Medical & Biological Laboratories Co. Ltd., Tokyo, Japan), or anti‐p63 (ab97865, Abcam, 1:500), or anti‐PCNA (SC‐56, Santa‐Cruz, Dallas, TX, USA, 1:200), or anti‐cleaved caspase‐3 (Asp175) (9661 s, Cell Signaling Technology 1:200), or anti‐E‐cadherin (610,181, BD Bioscience, Franklin Lakes, NJ, 1:200) in blocking solution overnight at 4°C. Stained sections were washed with 0.01% Triton in PBS (PBST 0.01%) for 10 min thrice, followed by secondary antibodies goat anti‐chicken IgY AF488 (A11039, Invitrogen), or goat anti‐mouse IgG AF647 (A32728, Invitrogen), or goat anti‐mouse IgG AF488 (A11029, Invitrogen), or goat anti‐rabbit IgG AF488 (A11034, Invitrogen), or goat anti‐rabbit IgG AF647 (4414, Cell Signaling Technology, Danvers, MA, USA) 1:500, and nuclear staining with Hoechst 33342 (H3570, Thermo Fisher Scientific) 1:500 for 1.5 h at RT. Sections were washed with PBST 0.01% for 10 min, 3 times, followed by PBS rinse thrice and mounted using VectashieldVibrance (Cat no. H‐1700, VectorLab, Newark, CA, USA).

### 4.12 Mouse Tissue Sectioning and Immunochemistry

Mouse tail skin specimens were immersed in ice‐cold 4% paraformaldehyde (4% PFA), irradiated in a 500‐W microwave oven for 3 × 30‐s cycles with intervals, and then kept on ice for 20 min. The fixed skin samples were embedded in optimal cutting temperature (OCT) compound (Sakura Finetechnical), snap‐frozen in liquid nitrogen, and then stored at −80°C. Frozen samples were cut into 10‐μm‐thick sections using a CryoStar NX50 cryostat (Thermo Fisher Scientific). Prior to staining, stored frozen sections were air‐dried at RT for 1 h and then rinsed with PBS for 5 min thrice, followed by blocking with 3% skim milk (Difco) (BD 232100, Thermo Fisher Scientific) and 0.1% Triton X‐100 for 30 min at RT. Sections were then incubated with primary antibody anti‐IRE1 (phospho S724) (ab48187, Abcam, 1:200) in blocking solution overnight at 4°C. Stained sections were washed with PBS (PBST, 0.01%) for 5 min thrice, followed by incubation with secondary antibody goat anti‐rabbit IgG AF488 (A11034, Invitrogen) 1:500 for 2 h at RT and nuclear staining with Hoechst 33342 (H3570, Thermo Fisher Scientific) 1:500 for 20 min at RT. Sections were then washed with PBS for 5 min thrice and mounted using VectashieldVibrance (Cat no. H‐1700, VectorLab, Newark, CA, USA).

### 4.13 Proteostat Staining

Protein aggregates were visualized with the Proteostat aggresome fluorescent staining kit (Cat no. ENZ‐51035 Enzo Biochem Inc., NY, USA). Air‐dried sections were washed with 0.01% Triton in PBS (PBST 0.01%) for 10 min three times. Dye was diluted 1:500 in PBS and incubated for 10 min at RT, followed by washing in PBS for 5 min, three times. The sections were destained in 1% acetic acid for 30 min at RT, followed by immunostaining as described above.

### 4.14 Transmission Electron Microscopy

Livers were collected and fixed using 4% formaldehyde in 0.1 M phosphate buffer (pH 7.4) for 1 h at RT and then washed using 0.1 M phosphate buffer (pH 7.4) containing 4% sucrose for 15 min, 3 times, and soaked in 10%, 15%, and 20% sucrose in 0.1 M phosphate buffer for cryoprotection, followed by a 5 min incubation in O.C.T. compound and then flash frozen in liquid nitrogen and stored at −80°C. Cryosectioning was then performed as described previously with around 10–20 μm thickness, and samples were transferred to 12 mm MAS‐coated micro‐cover glass (Matsunami Glass Ind. Ltd., Kishiwada, Japan) and allowed to dry for 30 min. Tissue sections on cover glass were soaked in 0.1 M phosphate buffer (pH 7.4), post‐fixed in 1% OsO_4_ and 1% potassium ferrocyanide in 0.1 M phosphate buffer (pH 7.4) for 1 h at room temperature, and washed in H2O. The specimens were dehydrated in a graded series of ethanol and embedded in epoxy resin for 2 d at 60°C. Ultrathin sections were cut and stained with uranyl acetate and Pb solutions. Electron micrographs were obtained using a VELETA, 2 K × 2 K side‐mounted TEM CCD camera on a JEM 1400 plus (JEOL, Akishima, Japan) transmission electron microscope at 80 kV.

### 4.15 Cell Treatment and Cell Count Analysis

Cells were seeded in 6‐well plates at 2 × 10^5^ cells/well, and at least 20 h after seeding, cells were treated for 4 and 5 days with 4μ8C, NMS‐873 (Cat no. S7285, Selleck Chemicals), or DMSO. Cells were collected for RNA extraction or cell counting in 1:1 0.4% trypan blue solution (Cat no. 72–57‐1, Sigma‐Aldrich). For tunicamycin treatment, cells were seeded at 1 × 10^5^ cells/well and treated with 5 mg/mL tunicamycin or DMSO for at least 20 h after seeding for 4 h, followed by a PBS wash and medium change. Cells were collected and counted at 4 days post‐treatment (dpt). For glucose treatment, cells were seeded at 2 × 10^5^ cells/well for RNA extraction and 1 × 10^5^ cells/well for cell counting in no‐glucose DMEM (Cat no. 08459, Nacalai Tesque Inc.) for 24 h. For RNA extraction, the medium was then changed to high‐glucose DMEM (Cat no. 09891, Nacalai Tesque Inc.), and after 0, 15, 30, and 60 min, cells were washed using PBS, and RNA was extracted for qPCR. For cell count analysis, the medium was changed to high‐glucose DMEM (Cat no. 09891, Nacalai Tesque Inc.), followed by cell counting at 7 dpt.

### 4.16 Cell Treatment and Immunochemistry

For cell death analysis, cells were seeded at 2 × 10^5^ cells/well in 6‐well plates for 24 h followed by medium change containing DMSO, Tunicamycin, 4μ8C, NMS‐873, or no‐glucose DMEM for 5 days. Doxorubicin HCl (040‐21, 521, FUJIFILM Wako Pure Chemical Co.) treatment for 2 days was used as a positive control for cell death. After treatment, cells were washed in PBS followed by fixation in 4% PFA in PBS for 15 min, and then washed with PBS, PBST 0.1%, and PBS for 5, 20, and 5 min, respectively, followed by blocking with 3% FBS in PBS for 1 h at 4°C and first antibody staining using anti‐cleaved caspase‐3 (Asp175) (9661 s, Cell Signaling Technology) 1:4000 in PBS overnight at 4°C. Wells were washed with PBS thrice for 5 min each followed by secondary antibody staining using goat anti‐rabbit IgG AF488 1:4000 for 2 h at RT, and nuclear staining with Hoechst 33342 1:4000 for 20 min at RT. Wells were washed with PBS twice for 5 min followed by imaging using a water immersion lens. For PCNA analysis, cells were seeded at 2 × 10^5^ cells/well on a cover glass inside a 6‐well plate for 24 h followed by tunicamycin treatment for 4 h, or 4μ8C, or NMS‐873 as described above. Cells were then washed in PBS and fixed using 4% PFA in PBS for 15 min. Cover glasses were washed using PBS and transferred to cover glass staining jars for antigen retrieval using Dako target retrieval solution, citrate pH 6 at 95°C for 10 min followed by PBS and PBST 0.1% wash and antibody staining as described above.

### 4.17 *N. furzeri* Treatment

For tunicamycin, animals were submerged in either 1 μg/mL tunicamycin or DMSO diluted in system water, placed in separate containers for 24 h, and then followed by tissue collection as described previously. For NMS‐873, 2‐week‐old fish larvae were submerged in either 2.5 nM NMS‐873 or DMSO diluted in system water, placed in separate containers for 4 days, followed by a medium change daily with brine shrimp given once every day, and larvae were collected and fixed as described previously.

### 4.18 Generation and Confirmation of Ire1 Knockout Embryos

The sgRNA targets listed in Table S2 were retrieved from NCBI Gene IDs (*ern1*:107376527, *ern2*:107388775). To induce frameshift mutations, target sites were selected using the chop‐chop program based on protein‐coding sequences for each gene that did not overlap with other genomic sequences. sgRNA and oligonucleotide synthesis, injections at the 1‐cell stage, and DNA digestion efficiency were performed as previously described (Oginuma et al. 2022). RNA expression of *ern1* and *ern2* was confirmed using qRT‐PCR as described above.

### 4.19 Photo‐Isolation Chemistry Spatial Transcriptome Analysis

Aged *N. furzeri* were treated with tunicamycin, whereas control young and aged *N. furzeri* were treated with DMSO, as described above. *N. furzeri* trunk and fin were fixed and sectioned as described above. Spatial transcriptome analysis using photo‐isolation chemistry was performed as previously described (Honda et al. 2021). Sections were permeabilized in TE buffer preheated to 70°C for 1 h in a staining jar and then cooled down in PBS for 3 min. Reverse transcription was performed by adding UV‐responsive 6‐nitropiperonyloxymethyl‐caged reverse transcription primers containing a T7 promoter, unique molecular identifiers (UMIs), multiple barcodes, and a poly T sequence to the trunk and fin sections. Immunofluorescence for p63 was performed as described above to visualize the epidermal basal layer after UV irradiation. p63‐positive cells in the epidermal basal layer were irradiated with UV light for 3 min using a Digital Micromirror Device (Polygon 1000‐G, Mightex) to uncage 6‐nitropiperonyloxymethyl moieties from the reverse transcription primers. Total tissue lysates were collected and purified using 20 mg/mL proteinase K. Second‐strand DNA was synthesized using the nick translation method. The synthesized cDNAs were transcribed into RNAs using a T7 Transcription Kit in vitro. After the collection of epidermal basal layer‐specific cRNAs, the RNAs were reverse‐transcribed, followed by paired‐end sequencing on an Illumina platform (Read 1: UMIs and barcode, Read 2: cDNA). Sequences were separated by sample barcodes using UMI tools, mapped to the reference genome using HISAT2, and used to generate UMI count data assigned to the genes.

DEGs were extracted using DESeq2 (FDR = 0.5). DESeq2 was also used to transform the count data into regularized log data before performing PCA using the R‐prcomp function. Enrichment maps were constructed with Cytoscape 3.9 and the BiNGO plug‐in using the default settings. The degree of enrichment for each GO was assessed at *p* < 0.5. GSEA was performed on normalized counts of the RNA‐seq datasets. The GO enrichment analysis was performed using ShinyGO 0.77 (Ge et al. 2020). Clusters of functionally related enriched GO terms were identified manually.

### 4.20 Lactate Assay

Dorsal fins from 1.5 month to 4 month *N. furzeri* were lysed using a bio‐masher in 0.5% Tirton‐X in PBS, then centrifuged, and the supernatant was collected. Total protein was measured using the BCA assay in accordance with the Pierce BCA Protein Assay kit (Cat no. 23225, Thermo Fisher Scientific), followed by a lactate assay using the Lactate Assay Kit‐WST (Cat no. L256, Dojindo Laboratories Co. Ltd., Mashikimachi, Japan).

### 4.21 Glucose Assay

Dorsal and ventral fins from 1.5 month to 4 month *N. furzeri* were lysed using a bio‐masher in homogenization buffer according to the kit manufacturer, then centrifuged and the supernatant was collected, followed by a glucose assay using the Glucose‐Glo Assay (Cat no. J6021, Promega Co. Ltd., Madison, WI USA). Total protein was measured from the sample diluted in 0.5% Triton using the BCA assay in accordance with the Pierce BCA Protein Assay kit (Cat no. 23225, Thermo Fisher Scientific).

### 4.22 Microscopic Imaging and Image Analysis

Imaging was performed using an Olympus FW3000 confocal laser scanning microscope with SV‐3000 software (Olympus, Tokyo, Japan). The interval for each focal plane was 2 μm. For analysis of the epidermis, several images were captured from the dorsal and ventral fins, covering a total length of approximately 1–1.5 mm (approximately 20%–30% of the total fin length), measured from the trunk. Images in Figure S1E were captured using a fluorescence stereomicroscope M205FA with Las‐X software (Leica Microsystem GmbH, Wetzlar, Germany), and images in Figure S4A,B were captured using BZ‐X810 (Keyence, Osaka, Japan). Confocal images were captured in a single plane with either a 10× or 20× objective lens. The number of nuclei, PCNA‐ and p63‐positive cells, active caspase‐3‐ and FK1‐positive foci, and the intensity/area (μm^2^) of GFP and E‐cadherin levels were measured using ImageJ software. All the images were minimally processed using brightness and contrast adjustments. Adjustments were applied equally across the entire image and the controls. Regions of interest were selected by a segmented line based on the anatomical features of each region or were randomly selected.

### 4.23 Quantification and Statistical Analysis

All data are represented as mean ± s.d. Two‐tailed unpaired Student's *t*‐test was used to compare data between two independent groups, and Dunnett's multiple comparison ANOVA was used to compare data between more than two independent groups. All data were analyzed using the Prism 8 software (GraphPad). The *p* value was used to define the significance of the differences between the groups. Statistical *p* was set less than 0.05; the groups were identified as significantly different. The numbers of biological replicates are listed in the figure legends. *p* values or asterisks indicate differences in the figures.

## Author Contributions

Conception and design: D.S., K.Ab., and T.I. wrote the main manuscript text; D.S., K.Ab., M.H., H.O., K.As., E.K.N., S.O., Y.O., and T.I. analyzed the data; D.S., K.Ab., H.O., and T.I. prepared the figures. All authors have reviewed the manuscript.

## Conflicts of Interest

The authors declare no conflicts of interest.

## Supporting information


**Appendix S1:** acel70258‐sup‐0001‐AppendixS1.pdf.

## Data Availability

The data that support the findings of this study are openly available in GEO at https://www.ncbi.nlm.nih.gov/geo/, reference number GSE287914.
