# Supplementary material for: ER Stress Ire1‐Xbp1s Pathway Maintains Youthful Epidermal Basal Layer Through the Regulation of Cell Proliferation
Source: Aging Cell. 2025 Oct 12;24(12):e70258. doi: 10.1111/acel.70258 (PMC12686570; doi:10.1111/acel.70258)
Supplement: Supplementary file 1 — Appendix S1: acel70258‐sup‐0001‐AppendixS1.pdf. [file ACEL-24-e70258-s001.pdf]

Supporting Information

ER stress Ire1-Xbp1s pathway maintains youthful epidermal basal layer through the regulation of cell proliferation

- **Supplemental Data Figures S1-S8**
- **Supplemental Data Tables 1-2**

Supplemental Data Figures S1-S5

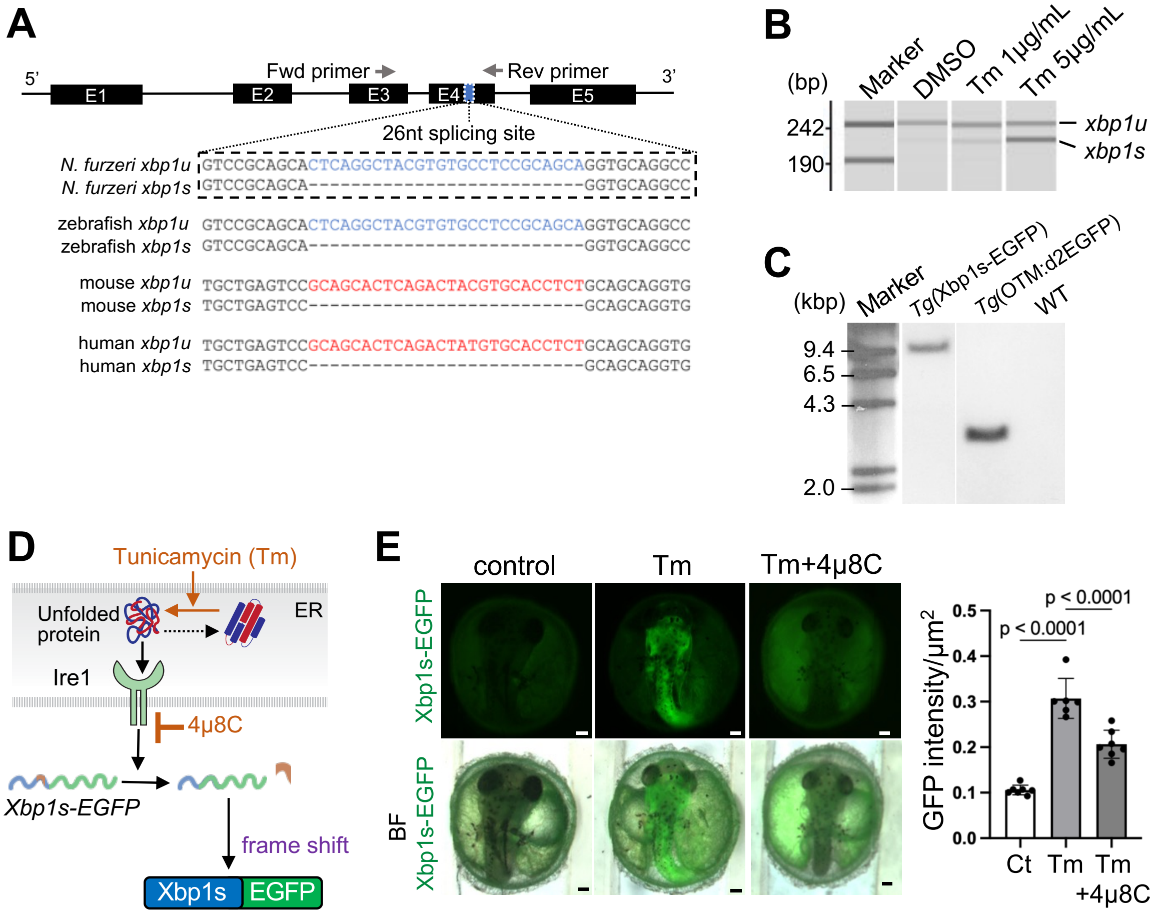

Figure S1. Establishment of Xbp1s-GFP reporter-transgenic *N. furzeri*.

(A) A 26-nt intron of *xbp1* transcript spliced out in *N. furzeri*, similar to that in zebrafish, mice and humans. The 26-nt *xbp1* intron sequence and its surrounding sequences in *N. furzeri* were identical to those in zebrafish.

(B) *xbp1* un-spliced (*xbp1u*) and spliced variant (*xbp1s*) from *N. furzeri* embryos after treatment with DMSO (control) or tunicamycin (Tm).

(C) Southern blot showing that Tg(xbp1s-GFP) carries a single-copy reporter. The established zebrafish reporter Tg(OTM:d2EGFP) and wild type *N. furzeri* (GRZ) were used as positive and negative controls, respectively.

(D) Schematic diagram of the action mechanisms of Tunicamycin and 4μ8C.

(E) Representative image and quantification of GFP intensity (E) in 10 days post fertilisation (dpf) embryos treated with DMSO and Tunicamycin (Tm) (5 μg/mL) without or with 4μ8C (2.5 mM). Scale bars, 100 μm. Values are mean ± s.d. (n=7, 6, 7); p = Dunnett's multiple comparisons of one-way ANOVA to tunicamycin-treated samples.

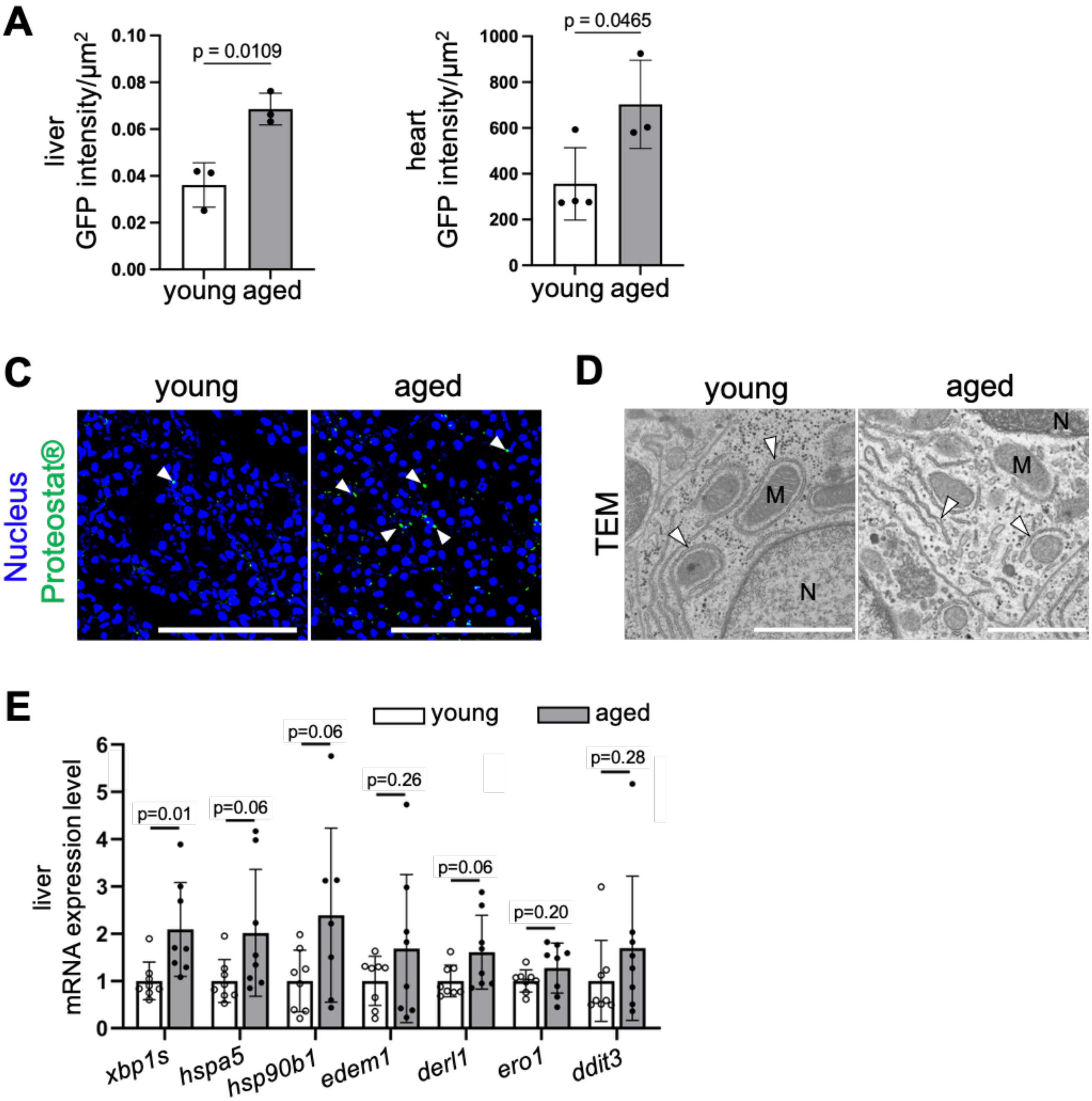

Figure S2. Chronic ER stress occurs in aged *N. furzeri* liver.

(A and B) Ire1-Xbp1s activity increases in the liver and heart with age. Quantification of GFP intensity in the liver (Figure 1C) (A) and heart (Figure 1D) (B). Values are mean ± s.d., $n = 3$ each for (A) and 4 and 3 for young and aged samples for (B); p = Student's *t*-test.

(C) Accumulation of unfolded proteins in the aged liver. Representative images of unfolded protein staining using Proteostat in the young and aged liver, white arrowheads show protein aggregates. Scale bars, 100 µm.

(D) ER fragmentation and expansion in the aged liver, suggesting ER stress. Representative images of transfer electron microscopy (TEM) of young and aged livers; white arrowheads show ER. Scale bars, 2 µm.

(E) Expression of ER stress-response target genes encoding chaperone proteins (*hspa5* and *hsp90b1*), ER degradation machinery proteins (*edem1* and *derl1*), ER oxidase (*ero1*), cell cycle arrest, and apoptosis-inducing transcription factor (*ddit3*) is upregulated in the aged liver. mRNA expression levels in young and aged *N. furzeri* are shown as fold-change relative to young *N. furzeri*. Values are mean ± s.d., n = 8 each; p = Student's t-test.

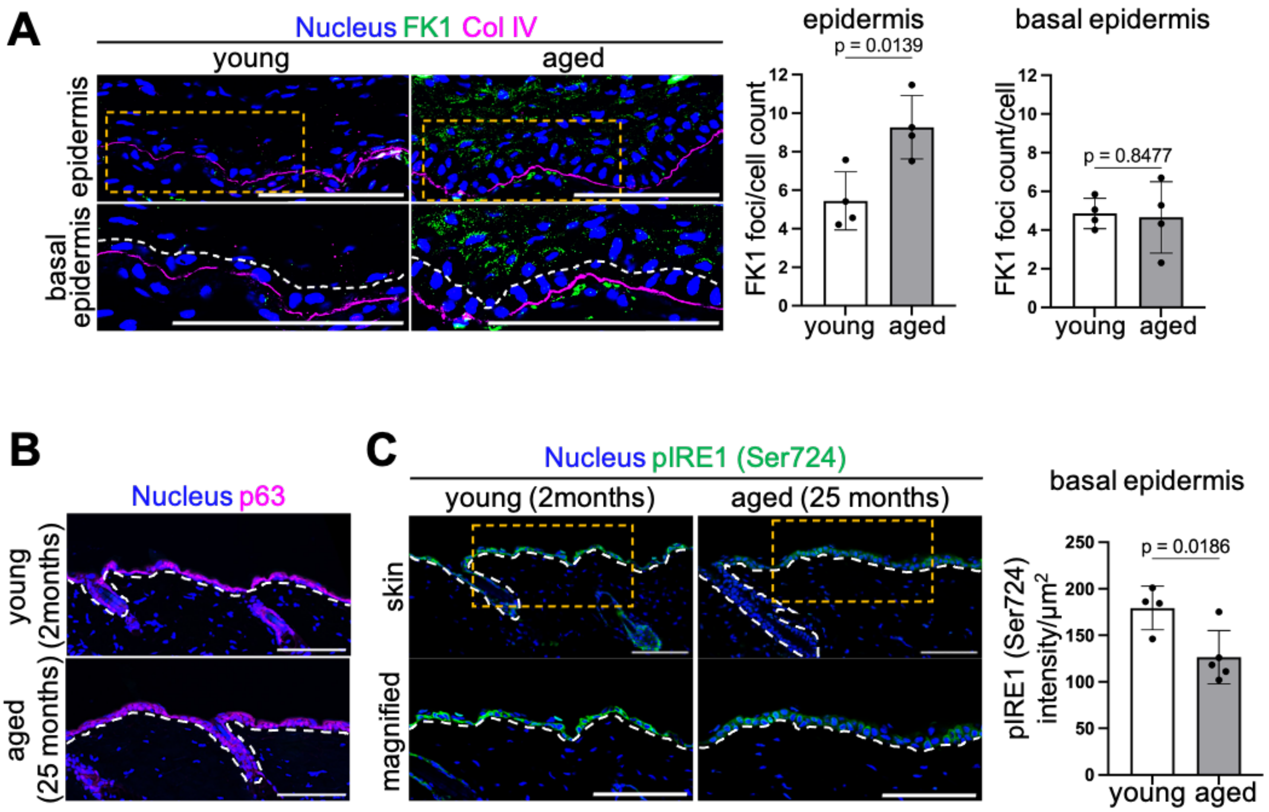

Figure S3. Unfolded protein accumulation in the epidermis of *N. furzeri* and the decline of Ire1-Xbp1s activity in the epidermal basal layer of aged mouse.

(A) Unfolded protein accumulates in the upper layer of aged epidermis, but not in the basal layer. Representative images of FK1 poly-ubiquitin staining in young (1.2 month) and aged (4 month) *N. furzeri* fin epidermis, and FK1 foci/cell count. Col IV (Collagen type IV) was used as a basement membrane marker. Yellow dashed lines indicate basal epidermis areas shown in the bottom panels. Scale bars, 100 µm. Values are mean ± s.d., *n* = 4; p = Student's *t*-test.

(B and C) The activity of Ire1-Xbp1s pathway in the basal layer of mouse epidermis declines with age, similar to that in *N. furzeri*. The location of epidermal basal layer in young (2 months) and aged (25 months) mouse tail epidermis was confirmed by anti-p63 staining (B). Representative images of anti-phospho-IRE1 (pIRE1) (Ser724) staining in young (2 months) and aged (25 months) mouse tail epidermis, and intensity/area (µm2) measurement (C). Yellow dashed lines indicate epidermis areas shown in the bottom panels and white dashed line indicate the border between epidermis and dermis. Scale bars, 100 µm. Values are mean ± s.d., *n* = 4 and 5 for young and aged samples; p = Student's *t*-test.

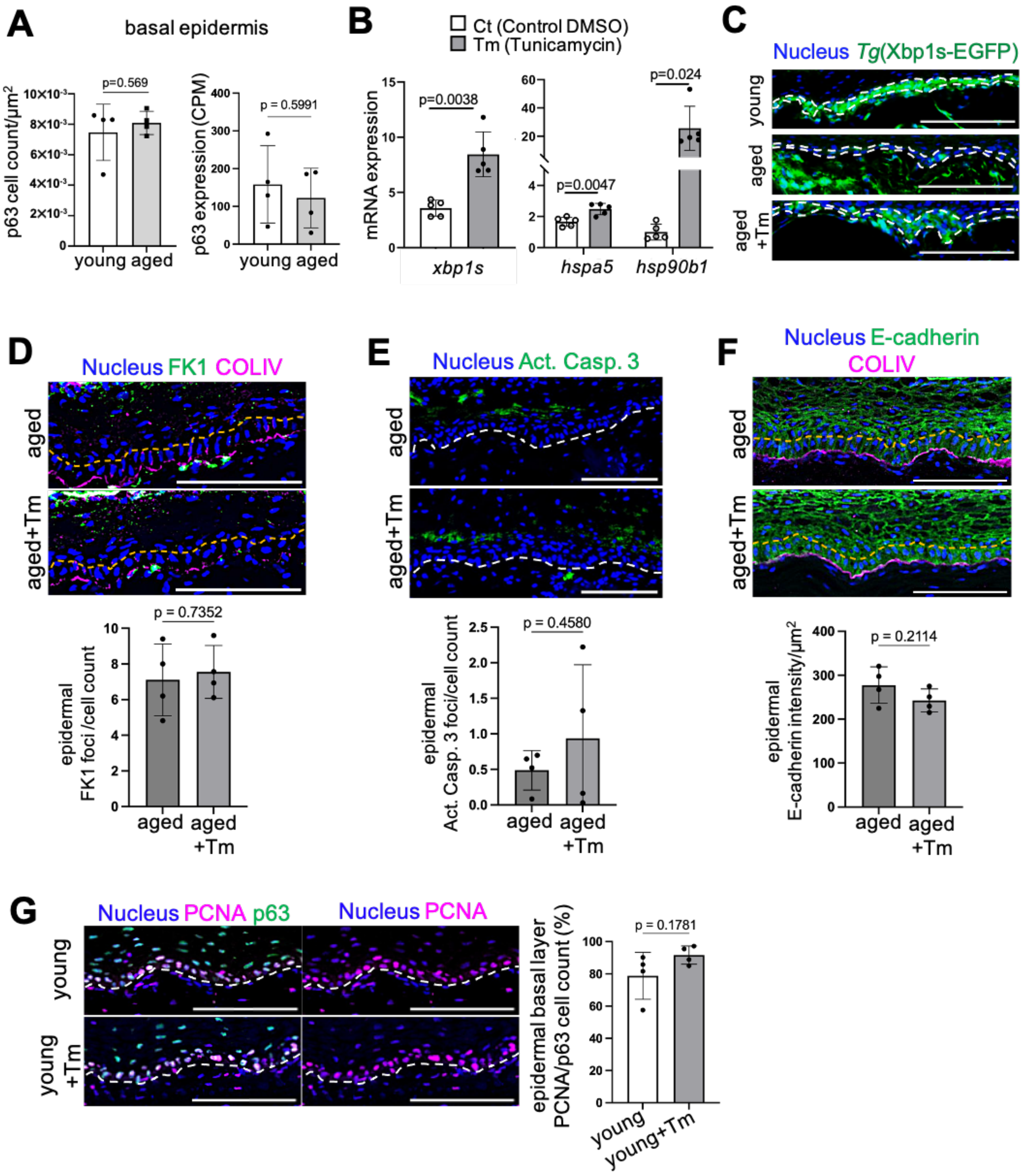

Figure S4. Forced activation and suppression of ER stress-response in *N. furzeri*.

(A) p63-positive cell number and p63 expression levels do not change in the basal layer of epidermis during aging. Cell count/area (100μm^2) of p63-positive cells (left) and expression level of *tp63* gene encoding p63 protein (right) in the young and aged basal layer epidermis. *tp63* expression data were

obtained from the PIC RNA-seq analysis. CPM = count per million; values are mean ± s.d., *n* = 4; p = Student's *t*-test.

(B) Transient treatment with tunicamycin increases endogenous mRNA levels of *xbp1s* and ER stress-response-related chaperones, *hspa5* and *hsp90b1* in the entire aged epidermis. The relative expressions are shown. Values are mean ± s.d., *n* = 5; p = Student's *t*-test.

(C) Transient treatment with tunicamycin increases Xbp1s-EGFP reporter activity in the aged epidermal basal layer. GFP expression in the epidermal basal layer of young, aged, and aged Tm fins in *Tg*(Xbp1s-GFP). Scale bars, 100 µm.

(D-F) Transient activation of ER stress-response did not induce significant damages to epidermal cells. Representative images of FK1 poly-ubiquitin staining and FK1 foci/cell count (D), active caspase 3 and foci/cell count (E), and E-cadherin and intensity measurement (F) in aged (4.2 month) and tunicamycin-treated aged (aged+Tm) *N. furzeri* fin epidermis. Yellow and white dashed lines indicate the border between supra-basal and basal epidermis, and epidermis and dermis, respectively. Scale bars, 100 µm. Values are mean ± s.d., *n* = 4; p = Student's *t*-test.

(G) Forced activation of ER stress-response in young *N. furzeri*. Representative images and quantification of PCNA-positive cells in young and tunicamycin-treated young (young Tm) *N. furzeri* fins normalized to p63. Scale bars, 100 µm. Values are mean ± s.d., *n* = 4; p = Student's *t*-test.

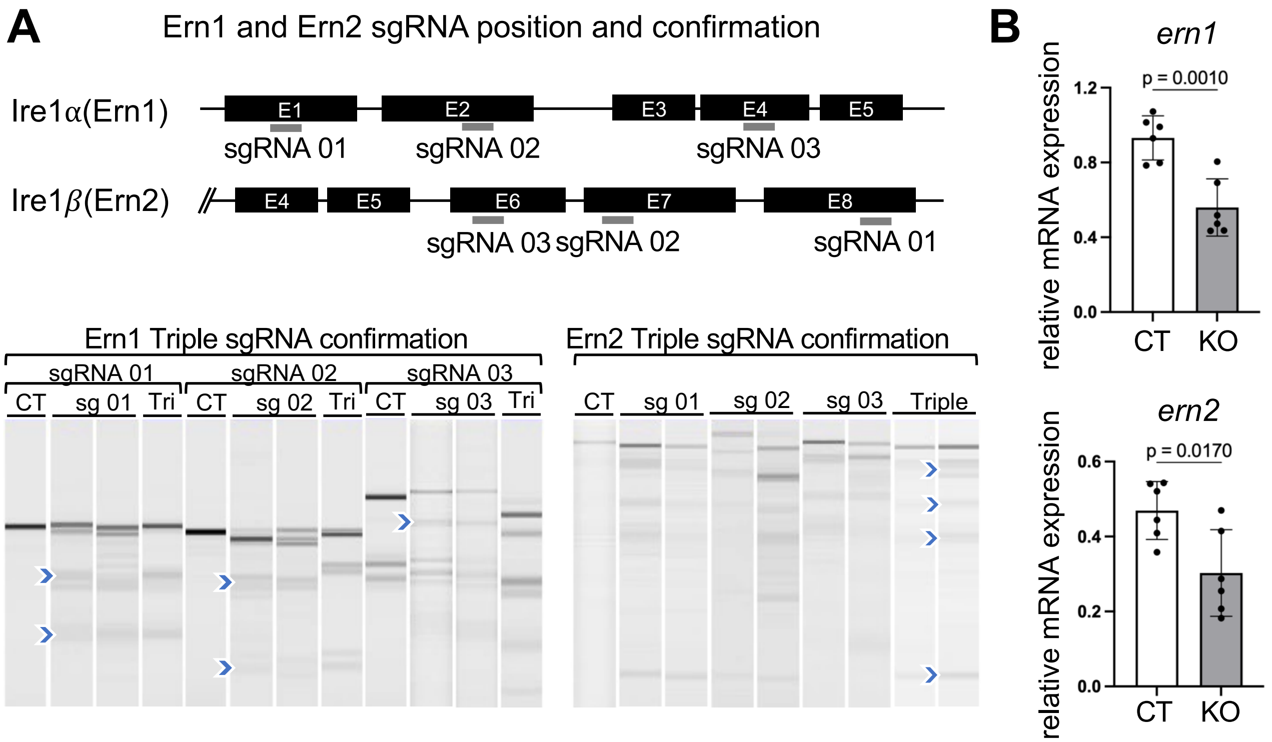

Figure S5. Triple-CRISPR-Cas9 KO of *ern1* and *ern2* genes in *N. furzeri*.

(A) Single guide RNA (sgRNA) design for triple CRISPR-Cas9 knock-out (KO) of Ire1α and Ire1β coding genes *ern1* and *ern2* and confirmation of single (sg 01, sg 02, and sg 03) and triple (Tri) *ern1* and *ern2* sgRNA compared to uninjected control (CT) in 5–7 days post injection embryos.

(B) Confirmation of mRNA expression of *ern1* and *ern2* in knock-out (KO) compared to control (CT) in 14 days post injection embryos. Values are mean ± s.d., $n = 6$; p = Student's t-test.

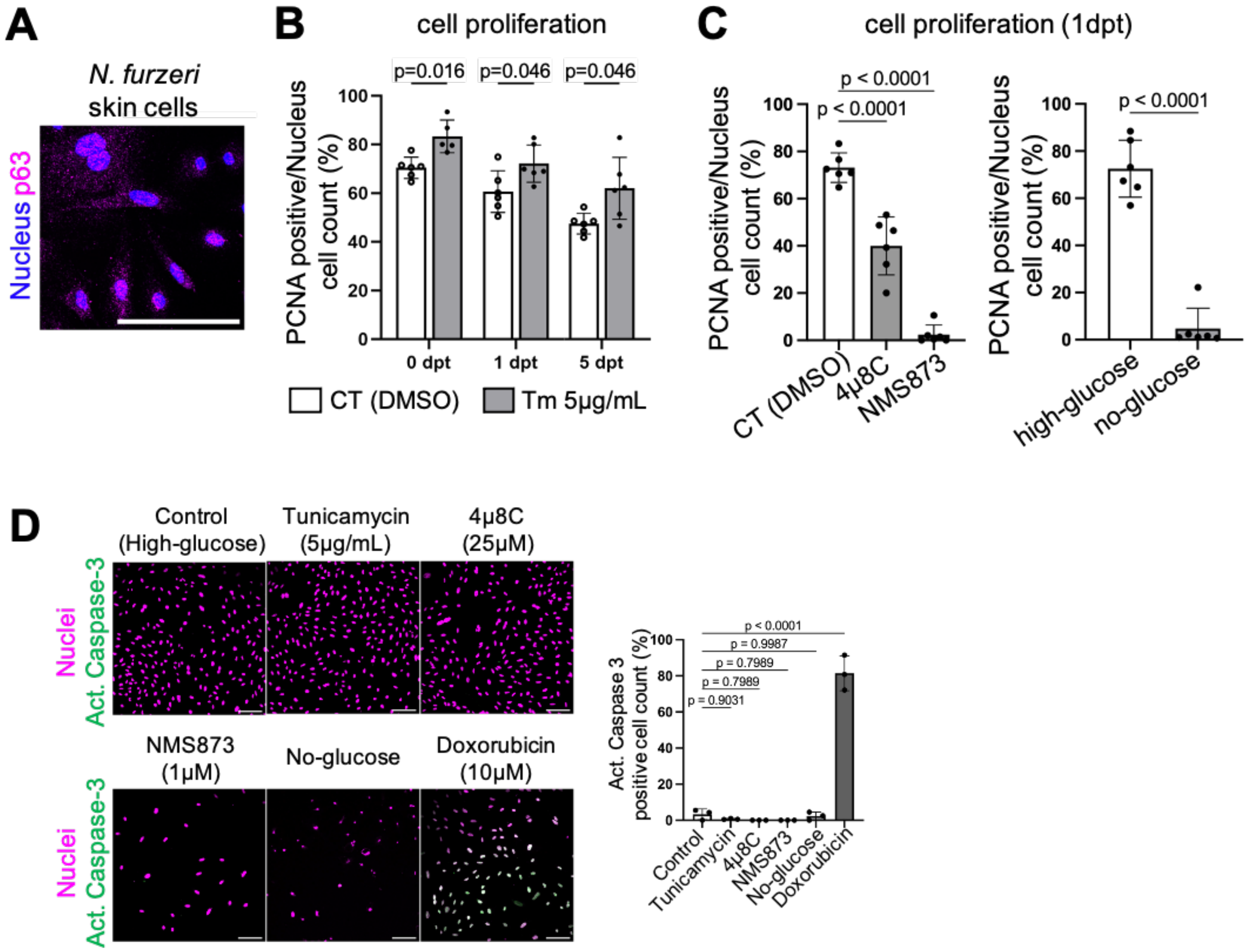

Figure S6. Confirmation of cell death and proliferation in drug-treated *N. furzeri* skin cells.

(A) *N. furzeri* epidermal cultured cells expressing p63 stem cell markers. Immunostaining for p63 protein. Scale bars, 100 µm.

(B) Glucose level change and treatment with tunicamycin, 4µ8C, or NMS873 does not induce apoptosis in *N. furzeri* skin cells. Active (Act.) caspase-3-positive cell counts of *N. furzeri* skin cells treated with high glucose (control), tunicamycin, 4µ8C, NMS873, no-glucose, or doxorubicin (cell death inducer). Scale bars, 100 µm; Values are mean ± s.d., *n* = 6; p = Dunnett's multiple comparisons of one-way ANOVA to control (high-glucose) sample.

(C) PCNA-positive cell counts of *N. furzeri* skin cells treated with tunicamycin at 0-, 1-, and 5-days post-treatment. Values are mean ± s.d., *n* = 6; p = multiple unpaired Student's *t*-test to control (DMSO) sample.

(D) PCNA positive cell count of *N. furzeri* skin cells treated with 4µ8C or NMS873 compared to control (CT) DMSO, and no-glucose treatment compared to high-glucose (4.5 g/L) at 1-day post-treatment. Values are mean ± s.d., *n* = 6; p = Dunnett's multiple comparisons of one-way ANOVA to control (DMSO) for 4µ8C or NMS873, and p = Student's *t*-test to high-glucose for no-glucose.

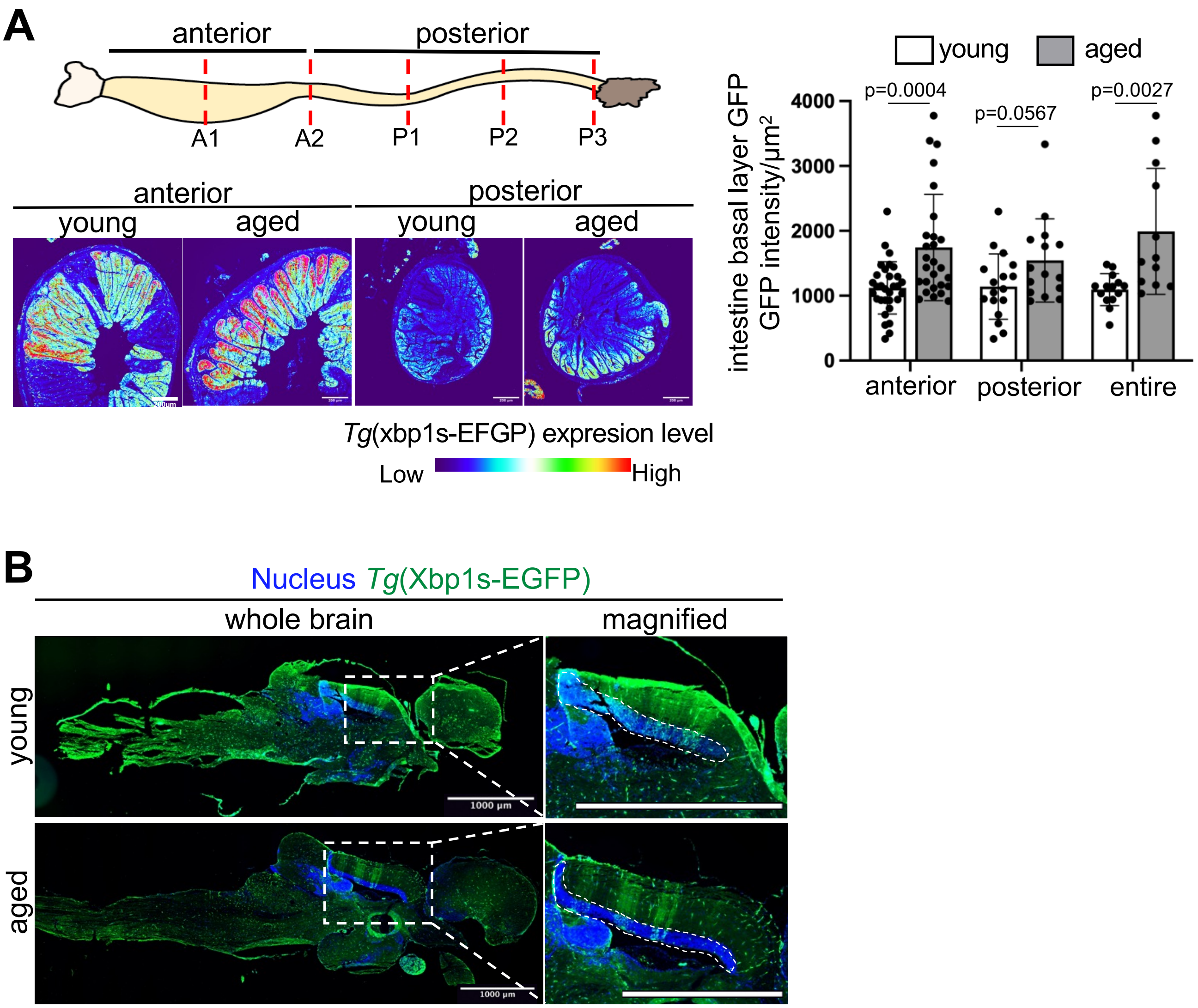

Figure S7. Age-dependent change of Ire1-Xbp1s activity in *N. furzeri* intestine and brain.

(A) Age-dependent activation of Ire1-Xbp1s activity in *N. furzeri* intestines. Representative images of GFP expression in anterior and posterior intestines of young and aged Tg(Xbp1s-GFP). Scale bars, 100 μm. Values are mean ± s.d., *n* (slice) = 17 and 15 (anterior), 14 and 19 (posterior), *N* (sample) = 5 and 4 for young and aged subjects, respectively; p = Student's *t*-test.

(B) Age-dependent inactivation of Ire1-Xbp1s activity in *N. furzeri* brain. Representative images of green fluorescent protein (GFP) expression in the brain and proliferative mesencephalic regions of young and aged *Tg*(Xbp1s-EGFP). Scale bars, 100 μm. N=2.

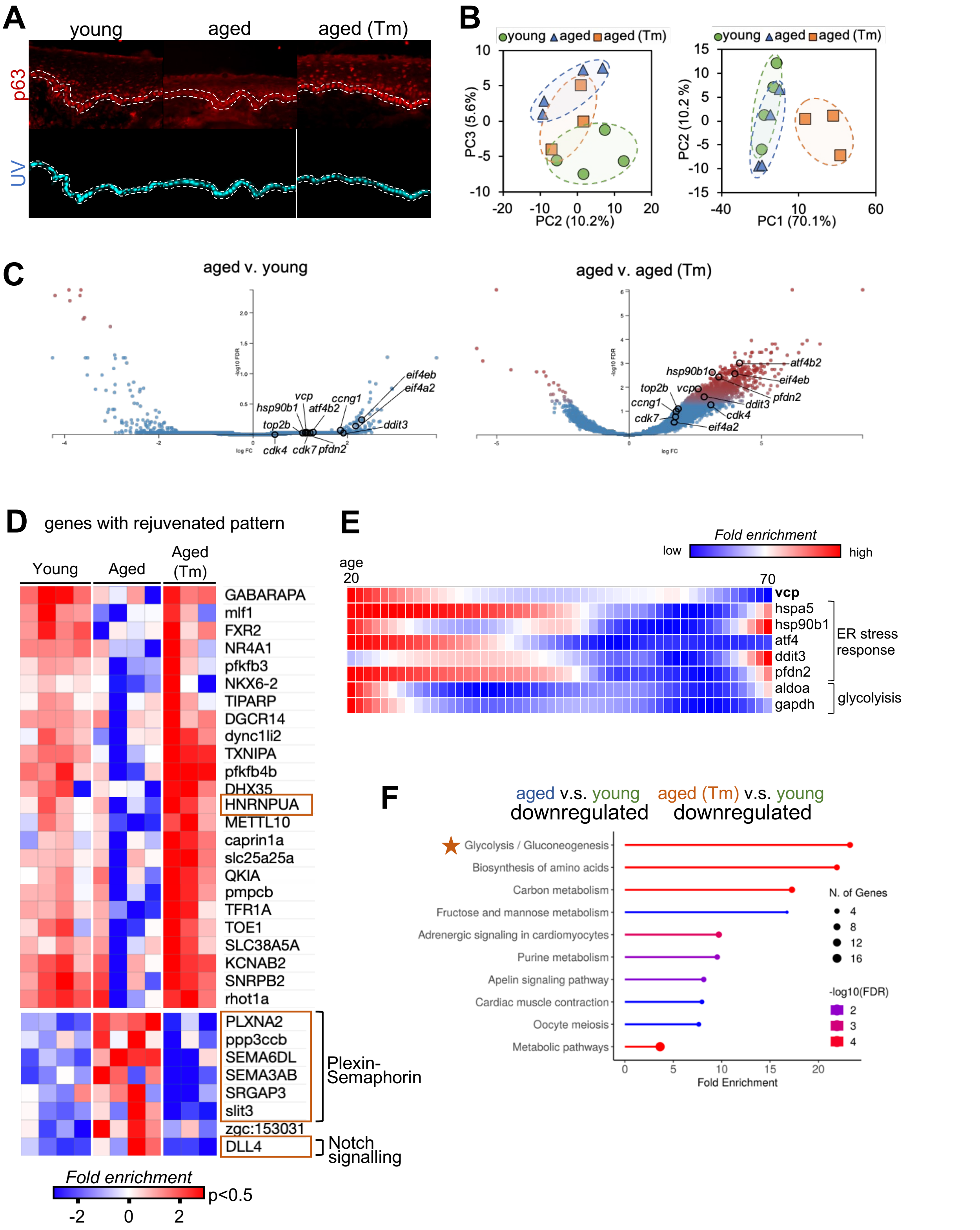

Figure S8. Spatial transcriptome using photo-isolation chemistry (PIC) and pathway analysis in the epidermal basal layer.

(A) p63 immunostaining and UV irradiation area in PIC samples.

(B) Transcriptomic patterns differ between young and aged epidermal stem cells, but tunicamycin treatment makes aged patterns closer to young ones. PCA analysis of the transcriptome in the basal layer of the fin showed genes that were rejuvenated to youthful conditions in tunicamycin-treated aged (aged Tm) *N. furzeri* in PC2 and PC3, whereas changes in gene expressions caused by cellular stress after tunicamycin treatment are shown in PC1 and PC2 analyses. n=4, 4, and 3 for young, aged, and aged samples, respectively (Tm).

(C) Volcano plot of DEGs, indicating genes that were shown in Figure 3B.

(D) Genes with rejuvenated pattern after tunicamycin treatment in aged killifish not related to ER stress response or cell cycle regulation. Upregulated genes include *hnrnpu*, and downregulated genes includes Plexin-Semaphorin (*plxna2*, *sema6dl*, and *sema3ab*) and Notch signalling (*dll4*) after tunicamycin treatment.

(E) Transcriptome of DEGs in human suprapubic skin. Similar to *N. furzeri,* the expression of *vcp*, ER stress-response-related genes, and glycolysis-related genes is downregulated with age in human skin.

(F) GO term analysis of genes that were downregulated in aged and aged Tm compared with young epidermal basal layer.

Table 1. List of primers used in this study.

Primer sequence (5′→3′)	Source	Name
**qPCR primers**		
TTCTGAGTCCGCAGCAGGTGCAGG	Eurofin	*xbp1s* F
GGCCCAGTAGCAAATCAG	Eurofin	*xbp1s* R
CGGTTGGAGGGTTTAGTCCT	Eurofin	*tbp* F
GCAAGACGATTCTGGGTTTG	Eurofin	*tbp* R
TTATCGTAAACGGTACTGGG	Eurofin	*hspa5* F
ATTCTGACTCATCACTGTCG	Eurofin	*hspa5* R
TACTATGCCAGCCAGAAGAA	Eurofin	*hsp90b1* F
CGTCTCAAACAGAACCACT	Eurofin	*hsp90b1* R
CCGTTCTATTTGCACGTTG	Eurofin	*edem1* F
CATGCGATCCTCTTTGGATT	Eurofin	*edem1* R
ATTATCTACCGTGGGTCATC	Eurofin	*derl1* F
ATGGGATACTTGTACATCAGG	Eurofin	*derl1* R
ACTGGCTTTCCTCAACATC	Eurofin	*ero1* F
TTCAATCTGTCGCTCTGAAA	Eurofin	*ero1* R
AGGATATTCTGGGCTCTGAT	Eurofin	*ddit3* F
ATGTCTGGTTTCCATCAGTC	Eurofin	*ddit3* R
ACTAACACATCTGCACTCTC	Thermo	*ern1* F
CCGAAGTCTGAGATCATAGC	Thermo	*ern1* R
CAAATGATCGATGCTGATGC	Thermo	*ern2* F
TCCTTCTCTATTCGGTCACT	Thermo	*ern2* R
***Tg(xbp1$^{[T]}$EGFP)* construct primers**		
GCGAGAGAAAACGAGCAGTC	Eurofin	endogenous *xbp1* F
GTCAGGACCATCCGTATCCA	Eurofin	endogenous *xbp1* R
GACGATGACGACAAGGGATCACGAGAGAAAACG AGCAGTCTG	Eurofin	flag site F
TGCTCACCATGTCAGGACCATCCGTATCC	Eurofin	flag site R
TGGTCCTGACATGGTGAGCAAGGGCGAGG	Eurofin	EGFP F
GAATTCGAATCGATGGGATCTTACTTGTACAGCT CGTCCATGCC	Eurofin	EGFP R
GCAGTCGACGCCACCATGGACTACAAAGACGAT GA	Eurofin	Tol2 OlActB *InFussion* F
GCAGTCGACTTACTTGTACAGCTCGTCCATGCC	Eurofin	Tol2 OlActB *InFussion* R
**Genotyping primers**		
CCCTTGCTCACCATGTCAGG	Eurofin	*Tg(xbp1$^{[T]}$EGFP)* F
GGGGATCGGGTCTTCTGAGT	Eurofin	*Tg(xbp1$^{[T]}$EGFP)* R

Table 2. List of CRISPR oligonucleotides and primers used in this study.

**Primer sequence (5′→3′)**	**Source**	**Name**
**CRISPR oligonucleotide**		
TAATACGACTCACTATAGGCGTGCAAGTTTCCAT CCAGTTTTAGAGCTAGAAATAGC	Eurofin	ERN1 *sgRNA01*
TAATACGACTCACTATAGGCAGGACCTGATTGCG CCCGTTTTAGAGCTAGAAATAGC	Eurofin	ERN1 *sgRNA02*
TAATACGACTCACTATAGGAATACAAGCTGCCAT CGTGTTTTAGAGCTAGAAATAGC	Eurofin	ERN1 *sgRNA03*
TAATACGACTCACTATAGGCCGAGTAATCGTTGT ACGGTTTTAGAGCTAGAAATAGC	Eurofin	ERN2 *sgRNA01*
TAATACGACTCACTATAGGACGATGCGTGGTTTG TAGGTTTTAGAGCTAGAAATAGC	Eurofin	ERN2 *sgRNA02*
TAATACGACTCACTATAGGCCACGTACAACGATT ACTGTTTTAGAGCTAGAAATAGC	Eurofin	ERN2 *sgRNA03*
**Confirmation primers**		
CTATGTAACCACCCACGGCTC	Eurofin	ERN1 *sgRNA01* conf F
CTACAGAACAGGGAACAGAGAGG	Eurofin	ERN1 *sgRNA01* conf R
CAGGTTTATCTAATCGCGCAGC	Eurofin	ERN1 *sgRNA02* conf F
GCTTTGCAGTGCCAGCAC	Eurofin	ERN1 *sgRNA02* conf R
GACTCAAAGAACCCTGAAAACCAA	Eurofin	ERN1 *sgRNA03* conf F
CCAGTTCTTCAGGTCCCCAC	Eurofin	ERN1 *sgRNA03* conf R
GTTCTGATGGCGTCCTTTATACAG	Eurofin	ERN2 confirmation F
CGTAGTTCTGAGTCCACAGGAC	Eurofin	ERN2 confirmation R
